# Universal 3D‐Printing of Suspended Metal Oxide Nanowire Arrays on MEMS for AI‐Optimized Combinatorial Gas Fingerprinting

**DOI:** 10.1002/advs.202511794

**Published:** 2025-08-26

**Authors:** Yu Liu, Kichul Lee, Hongjun Liu, Chenwei Li, Xiaoyi Zeng, Rongyue Liu, Yulong Chen, Zijun Chen, Jihyuk Yang, Xiao Huan, Inkyu Park, Ji Tae Kim, Xing Cheng

**Affiliations:** ^1^ The Greater Bay Area University Joint Laboratory of Micro‐ and Nanofabrication Department of Materials Science and Engineering Southern University of Science and Technology Shenzhen 518055 China; ^2^ Department of Mechanical Engineering The University of Hong Kong Pokfulam Road Hong Kong 999077 China; ^3^ Department of Mechanical Engineering Korea Advanced Institute of Science and Technology (KAIST) Daejeon 34141 Republic of Korea; ^4^ Advanced Materials Innovation Center Jiaxing Research Institute of Southern University of Science and Technology Jiaxing 314031 China; ^5^ Industrialization Center of Micro & Nano ICs and Devices Sino‐German College of Intelligent Manufacturing Shenzhen Technology University Shenzhen 518118 China

**Keywords:** 3D printings, convolutional neural network (CNN)‐based prediction, gas sensors, metal oxide nanowire

## Abstract

Additive manufacturing technology has the potential to provide great versatility in the design and fabrication of sensing devices. This prerequisite necessitates further technological improvement in precision and material diversity. Here, a universal meniscus‐guided 3D printing method is reported that can fabricate freestanding metal oxide semiconducting nanowires with programmed compositions and compatible substrate options at the single‐entity level. By studying printing process and ink compositions, polycrystalline metal oxide (MOX) nanowires with controlled shapes and tunable diameters down to 180 nm are achieved. The method enables a high‐precision, mask‐free printing of MOXs nanowire arches array on a 1.5 µm thick suspended membrane, paving the way for integrating a micro‐electromechanical systems (MEMS) chemiresistive gas sensor. The diversity of 3D printable materials demonstrated in this study covers 24 types of combination MOX nanowires, including TiO_2_, ZnO, SnO_2_, In_2_O_3_, WO_3_, and CeO_2_, doped with noble metals of Au, Ag, Pd, and Pt. Their sensing performances for CH_4_, NH_3_, CH_3_CH_2_OH, CO, and H_2_S gases are quantitatively investigated, while artificial intelligence (AI)‐driven analysis of multi‐sensor responses achieves 98% gas classification accuracy via sliding time window‐based convolutional neural networks (CNN). The ability to 3D print semiconducting materials opens the possibility to freely design and realize new‐concept electronic devices beyond the restrictions of the traditional top‐down manufacturing process.

## Graphical abstract

A universal meniscus‐guided 3D printing method enables direct fabrication of suspended metal oxide nanowires on MEMS chips. It achieves 180 nm resolution, compositional tunability, and integration on 1.5 µm thick membranes. Twenty‐four types of nanowires are printed to investigate gas responses, from which complementary materials enabled time window‐based CNN analysis with 98% classification accuracy. This strategy bridges 3D nanoprinting and practical electronic integration.

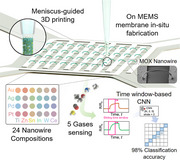

## 1 Introduction

3D printing is emerging as a disruptive technology that can transform the manufacturing industry.^[^ ^1^, ^2^, ^3^, ^4^ ^]^ Due to the unprecedented process simplicity and flexibility, 3D printing draws great attention from direct production in fields as diverse as biomedicine,^[^ ^5^, ^6^ ^]^ robotics,^[^ ^7^, ^8^, ^9^ ^]^ electronics,^[^ ^10^, ^11^, ^12^ ^]^ and photonics.^[^ ^13^, ^14^, ^15^ ^]^ Over the past decade, intensive research has been made to advance the precision and material in 3D printing technology. Recently, the meniscus‐guided method has been devised to improve the spatial resolution and material diversity that are not attainable in conventional approaches such as fused deposition modeling (FDM) and stereolithography apparatus (SLA). The method exploits a femtoliter ink meniscus formed on a printing nozzle to guide material assembly/crystallization in three dimensions, printing freestanding, complex nanostructures with a 100 nm spatial resolution.^[^ ^16^, ^17^, ^18^, ^19^ ^]^ Thanks to overwhelming compatibility with solution‐mediated material growth pathways, the method successfully demonstrated 3D printing of various functional materials such as metals,^[^ ^19^, ^20^, ^21^ ^]^ conducting polymers,^[^ ^18^, ^22^, ^23^, ^24^ ^]^ graphene/carbon nanotubes,^[^ ^25^, ^26^, ^27^, ^28^ ^]^ perovskites,^[^ ^29^, ^30^, ^31^ ^]^ metal‐organic frameworks,^[^ ^32^ ^]^ dipeptides,^[^ ^33^, ^34^ ^]^ and so on. Nevertheless, diversifying the material library remains a longstanding challenge in the field of 3D printing technology.

Metal oxide semiconductors have been of great interest to diverse fields due to their outstanding electrical and chemical properties.^[^ ^35^, ^36^, ^37^ ^]^ In particular, their 1D nanowires having a unique advantage of high surface area‐to‐volume ratio exhibit excellent performance in bio/chemical sensors,^[^ ^37^, ^38^, ^39^, ^40^ ^]^ supercapacitors,^[^ ^41^, ^42^, ^43^, ^44^ ^]^ batteries,^[^ ^45^, ^46^, ^47^ ^]^ catalysis,^[^ ^48^, ^49^, ^50^ ^]^ and photovoltaics.^[^ ^51^, ^52^ ^]^ Especially in the field of gas sensing, they show extraordinarily high sensitivity, fast response, and high tunability due to broader and more uniform interaction zones over cross‐section areas and confined carrier conduction paths along the long axis.^[^ ^53^, ^54^, ^55^, ^56^, ^57^, ^58^, ^59^ ^]^ Methods to prepare metal oxide nanowires have been extensively developed in two approaches: 1) bottom‐up chemical synthesis^[^ ^60^, ^61^, ^62^ ^]^ and 2) top‐down lithography.^[^ ^63^, ^64^ ^]^ The former provides high‐crystalline‐quality nanowires but continues to suffer from the stochastic nature of the resulting shape, size, and location.^[^ ^65^ ^]^ Integrating chemically synthesized metal oxide nanowires into circuitry inevitably necessitates additional labor‐intensive pick‐and‐place processes, hindering practicality.^[^ ^66^ ^]^ The latter provides a relatively easy integration route but involves destructive developing‐etching steps, leading to the degradation of material properties.^[^ ^67^, ^68^ ^]^ These technological challenges directly limit the fabrication of gas‐sensing microdevices.

Recently, additive manufacturing techniques have emerged as an alternative route for fabricating metal oxide gas sensor devices. Among them, multi‐photon polymerization is specialized for yielding high‐resolution metal oxide nanostructures, by utilizing specially designed photoresists that incorporate metal‐containing precursors, then, upon photopolymerization and subsequent pyrolysis.^[^ ^69^, ^70^ ^]^ However, the process occurs inside a liquid photoresist medium, rendering it challenging to be integrated with indispensable delicate micro‐electromechanical systems (MEMS) architectures,^[^ ^71^ ^]^ which are prone to collapse due to capillary forces or viscous stress of highly viscous photoresist. Therefore, new methods are still in great demand for direct, precise, and gentle fabrication of metal oxide nanowires on delicate circuit surfaces, achieving the practicality from 3D nanoprinting to electronic integration.^[^ ^72^ ^]^

In this work, we have developed a nanoscale 3D printing method that can directly fabricate metal oxide (MOX) nanowires with controlled shapes and compositions on a suspended membrane microheater, constructing a miniaturized gas sensor device. A key scheme is to exploit a femtoliter precursor ink meniscus produced by a micropipette to print nanowires with controlled diameters and lengths, followed by thermal calcination. The printed MOX nanowires exhibit a polycrystalline nature and are capable of gas sensing. The printing process is nondestructive and therefore can be readily carried out on a silicon nitride (Si_3_N_4_) and silicon oxide (SiO_2_) membrane with only a thickness of 1.5 µm suspended in a MEMS chip. It enabled the construction of a chemiresistive gas sensing microdevice configured with a MOX nanowire array integrated on an interdigitated electrode above a suspended microheater. The device with microscale local heating demonstrated a stable gas sensing operation at 300 °C with a power consumption of less than 40 mW. The method developed here is applicable to various metal oxides doped with different noble metals. The gas sensing performances of TiO_2_, ZnO, SnO_2_, In_2_O_3_, WO_3_, and CeO_2_ nanowires decorated with Au, Ag, Pd, and Pt for CH_4_, NH_3_, CH_3_CH_2_OH, CO, and H_2_S gases were scrutinized. By sliding time window‐based convolutional neural network (CNN) model, we achieved artificial intelligence (AI)‐optimized gas fingerprinting using combinatorial sensors, with the accuracy reached 98.0%. The 3D printing method is universally adaptable and shows high compatibility with other semiconducting processes, having strength in manufacturing heterogeneous devices. This would be a vital piece of the puzzle for the overall development of printed electronics.

## 2 Results and Discussion

### 2.1 Meniscus‐Guided 3D Printing of the Suspended MOX Nanowires

**Figure 1:**
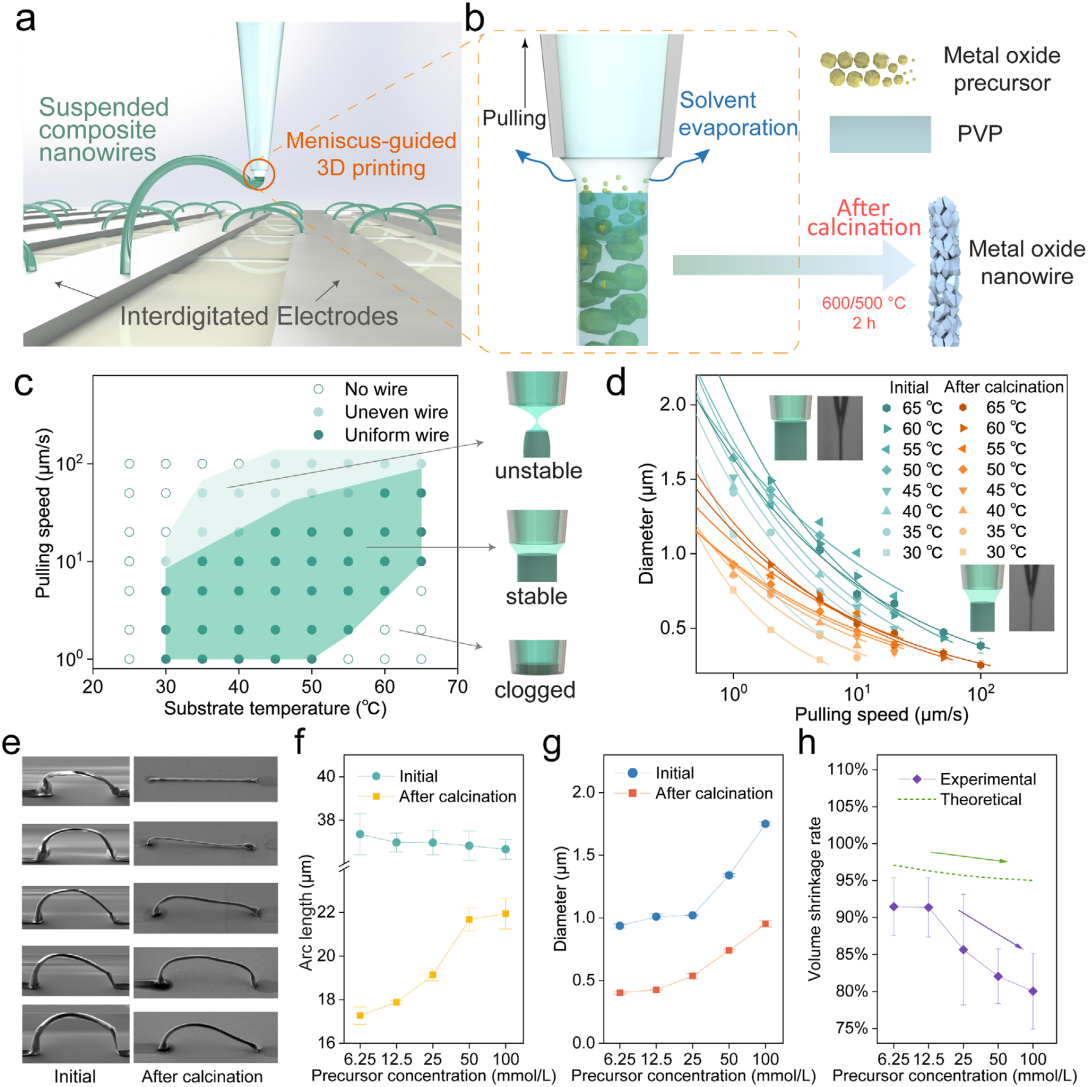
3D printing of the suspended MOX nanowire. a) Schematic illustrating the meniscus‐guided 3D printing of composite nanowires on interdigitated electrodes. b) Femtoliter meniscus guides continuous solidification along a vertical direction under solvent evaporation, followed by thermal calcination (600/500 °C, 2 h) for fabricating a polycrystalline MOX nanowire. c) Phase diagram displaying the dependence of the nanowire printability on the interplay between the pulling speed of a micropipette and the substrate temperature. Three corresponding states (unstable/stable/clogging) of the meniscus are shown on the right. d) Diameter of as‐printed (green) and calcinated (orange) nanowires versus pulling speed at different substrate temperatures ranging from 30 to 65 °C. e) Series of field‐emission scanning electron microscope (FE‐SEM) images showing as‐printed and calcinated nanowires with increasing precursor concentrations of 6.25, 12.5, 25, 50, and 100 mmol L^−1^, from the top to bottom, respectively. (scale bar: 5 µm). f) Dependence of nanowire arc length on precursor concentration at as‐printed (circles) and calcinated (squares). g) Dependence of nanowire diameter on precursor concentration at as‐printed (circles) and calcinated (squares). h) Volume shrinkage rate of the printed nanowire versus precursor concentration after calcination. Experimental (purple diamonds) and theoretical (dashed green line) plots.

**Figure** **1**a depicts the meniscus‐guided 3D printing of suspended composite nanowires. By guiding the printing position, the suspended bending of the nanowires can be achieved and connected to the interdigitated electrodes (IDEs). Figure 1b depicts how a polycrystalline metal oxide nanowire is produced during the printing process. A femtoliter‐volume meniscus of the ink comprising metal precursor and polyvinylpyrrolidone (PVP) is formed by contacting an ink‐filled micropipette to the substrate surface. Under the evaporative loss of solvent, the meniscus is rapidly solidified, leading to the formation of a precursor‐PVP composite. In this composite, PVP acts as a sacrificial structural binder, providing the necessary mechanical stability to the filament. Continuous meniscus guiding by pulling the pipette produces a freestanding composite nanowire. After the calcination process, the metal precursor transforms into MOX nanocrystals, and PVP is removed, resulting in a polycrystalline MOX nanowire. The printing and calcination processes were quantitatively examined using titanium di‐isopropoxide bis(acetylacetonate) (TAA) as the Ti precursor.

A diagram in Figure 1c shows the dependence of printability on the interplay between pulling speed and substrate temperature. The solid dark green circles indicate the successful printing of a uniform nanowire, whereas the hollow circles indicate failure. The solid light green circles mark unstable printing. Continuous, stable meniscus‐guided printing necessitates a balance between the solidification rate and the pulling speed (Figure 1c, insert, stable). When the pulling is much faster than the solidification, the meniscus ruptures, leading to a failure (Figure 1c, insert, unstable). Contrarily, excessive rapid solidification may lead to pipette clogging (Figure 1c, insert, clogging). The solidification rate of the meniscus by solvent evaporation is temperature‐dependent. The failure cases at low substrate temperatures below 30 °C and high pulling speeds are mainly attributed to insufficient solidification. The failures at high temperatures and low pulling speeds are mainly caused by pipette clogging. The regime for success printing is governed by two key criteria as schematically illustrated in Figure S1 (Supporting Information): i) the vectorial balance of interfacial tensions at the three‐phase contact line on the growing front following the Neumann quadrilateral relation and ii) the mass balance between the solvent evaporation‐induced solidification rate and the pipette pulling speed, determining the printing diameter (A detailed description is included in Figure S1, Supporting Information). The plots in Figure 1d show the dependence of the printed diameter on pulling speed in successful printing conditions. A general trend is that the printed diameter decreases to 500 nm as the pulling speed increases to 100 µm s^−1^ due to mechanical stretching of the meniscus. Meanwhile, it is also observed that raising the substrate temperature from 30 to 65 °C increases the diameter due to enhanced solidification. These trends follow the mechanism depicted in Figure S1 (Supporting Information), where within the stable meniscus angle window: 1) at constant mass rate, higher pulling speeds reduce the nanowire diameter; and 2) elevated substrate temperatures increase the mass rate, leading to thicker nanowires. The calcination process leads to a drastic decrease in the printed diameter. As shown in a clear distinction between green and orange curves in Figure 1d, the diameter shrinks by 40% after calcination. The resulting diameter of the calcinated nanowire ranges from 1 µm down to 180 nm (as shown in Figure S2, Supporting Information), under the successful printing window.

The volumetric shrinkage by calcination significantly affects the printed nanowire's length and diameter, as quantitatively investigated in Figure 1e–h. This volume shrinkage arises from the calcination, which was performed at 600 °C for 2 h to remove PVP with a molecular weight of 1300k, as the thermogravimetric analysis (TGA) result showed ≈ 99.5% elimination (Figure S3, Supporting Information). Such treatment ensures a high purity of metal oxide phase while minimizing residual PVP, which is critical for nanowire integrity and gas sensing behavior. The calcination temperature may be lowered if lower molecular weight PVP is used (Figure S4, Supporting Information). The nanowire's dimension and shape are directly influenced by the amount of precursor, as well as the ink composition that determines the shrinkage ratio. The field‐emission scanning electron microscope (FE‐SEM) images in Figure 1e show the calcination‐driven shape changes of the printed nanoarches (left: as‐printed, right: calcinated) prepared at different concentrations of the Ti precursor of 6.25, 12.5, 25, 50, and 100 mmol L^−1^ (from top to bottom). Overall, the shape change is suppressed as the precursor concentration increases.

Quantitatively, the shrinkages of the nanowire's arc length and diameter as a function of the precursor concentration are plotted in Figure 1f,g, respectively. The calcination‐driven shrinkage in arc length is significantly suppressed by increasing the precursor concentration (Figure 1f). The arc length was measured from the 45°‐tilted FE‐SEM images. Figure 1g shows the dependence of the diameter on the precursor concentration, obtained at a constant pulling speed of 10 µm s^−1^ and printing temperature of 45 °C. The diameter of the as‐printed nanowire increases as the concentration of the Ti precursor increases due to enhanced solidification (circled in blue). The calcination process decreases the diameter while maintaining a similar diameter‐concentration trend (squares in red). Overall, Figure 1h plots the volume shrinkage rate as a function of the precursor concentration. The experimental volume shrinkage rate (purple diamonds) was calculated as (1 – *V*_sintered_ / *V*_as‐printed_), where the volumes were estimated from the measured dimensions of the nanowires before and after calcination. The theoretical shrinkage rate (green dashed line), in contrast, was calculated based on the ink's solid content as (1 – *V*_MOXNW_ / *V*_compositeNW_), where *V*_MOXNW_ represents the ideal volume of the final, fully dense metal oxide and *V*_compositeNW_ is the initial volume of the precursor–PVP composite, with these volumes being estimated from the mass fractions of the components in the ink and their respective material densities. Both rates decrease as the precursor concentration increases, with the experimental values consistently below the theoretical ideal. This deviation is attributed to the non‐ideal nanostructure of the calcined nanowires. While the theoretical model assumes a perfect conversion to a fully dense, non‐porous solid, the actual resulting nanowires are polycrystalline and possess significant surface roughness and internal defects (such as voids). As the precursor concentration increases, these non‐ideal characteristics become more pronounced, resulting in a larger final nanowire volume (*V*_sintered_) than theoretically predicted. This retention of a larger final volume leads to a lower calculated shrinkage rate, explaining both the deviation from the theoretical value and the decreasing trend with higher precursor concentration. To fabricate freestanding nanowires with an effectively small diameter, a precursor concentration of 25 mmol L^−1^ and 35 µm length was primarily used in this study. The nanowire printing spanned over 500  µm and 1.1 mm in length while remaining intact after calcination, as demonstrated in Figure S5 (Supporting Information). The maximum achievable span is determined by the travel range of the motorized stage and the mechanical stability of the suspended nanowire. The result highlights excellent dimensional compatibility of our nanoprinting technique with MEMS applications, as 500  µm length exceeds the lateral dimension of most MEMS chips' suspension parts.

**Figure 2:**
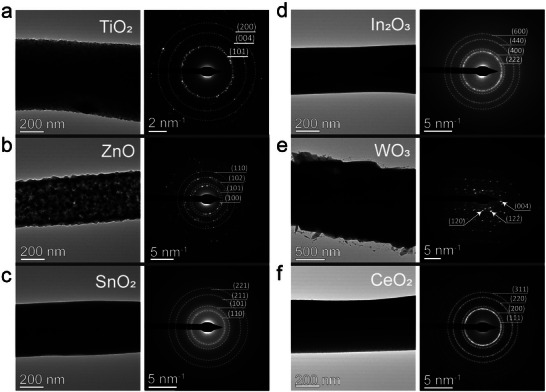
Characterization of printed MOX nanowires, including bright‐field transmission electron microscopy (TEM) images, and selected‐area electron diffraction (SAED) patterns of a) TiO_2_, b) ZnO, c) SnO_2_, d) In_2_O_3_, e) WO_3_, f) CeO_2_.

Our 3D printing approach demonstrates broad applicability across various MOX compositions simply by precursor substitution. With maintaining the same concentration of ink (25 mmol L^−1^) and processing conditions, we successfully fabricated 6 types of MOX nanowires, such as TiO_2_, ZnO, SnO_2_, In_2_O_3_, WO_3_, and CeO_2_. For crystallographic characterization, the nanowires were directly printed across a Si TEM grid having a 200 µm gap (Figure S6, Supporting Information). The polycrystalline nature of the printed MOX nanowires was confirmed by selected‐area electron diffraction (SAED) patterns and bright‐field transmission electron microscope (TEM) images (**Figure** **2** and Figure S7, Supporting Information). For example, the SAED pattern in Figure 2a contains distinct diffraction rings corresponding to the (101), (004), and (200) planes of anatase TiO_2_. The corresponding SAED patterns of polycrystalline ZnO, SnO_2_, In_2_O_3_, WO_3_, and CeO_2_ nanowires are shown in Figure 2b–f, respectively. The resulting diameters range from 400 to 600 nm, and the observed diameter variation could potentially result from subtle differences in the density and porosity of the printed MOXs. The larger diameter observed for WO_3_ nanowires (Figure 2e) may be attributed to the significantly higher molecular weight of its precursor (ammonium metatungstate hydrate, molecular weight: 2956.3) compared to others, which likely promoted enhanced polycrystallinity and porosity formation under identical calcination parameters.

### 2.2 Direct Printing of MOX Nanowire Array on MEMS

**Figure 3:**
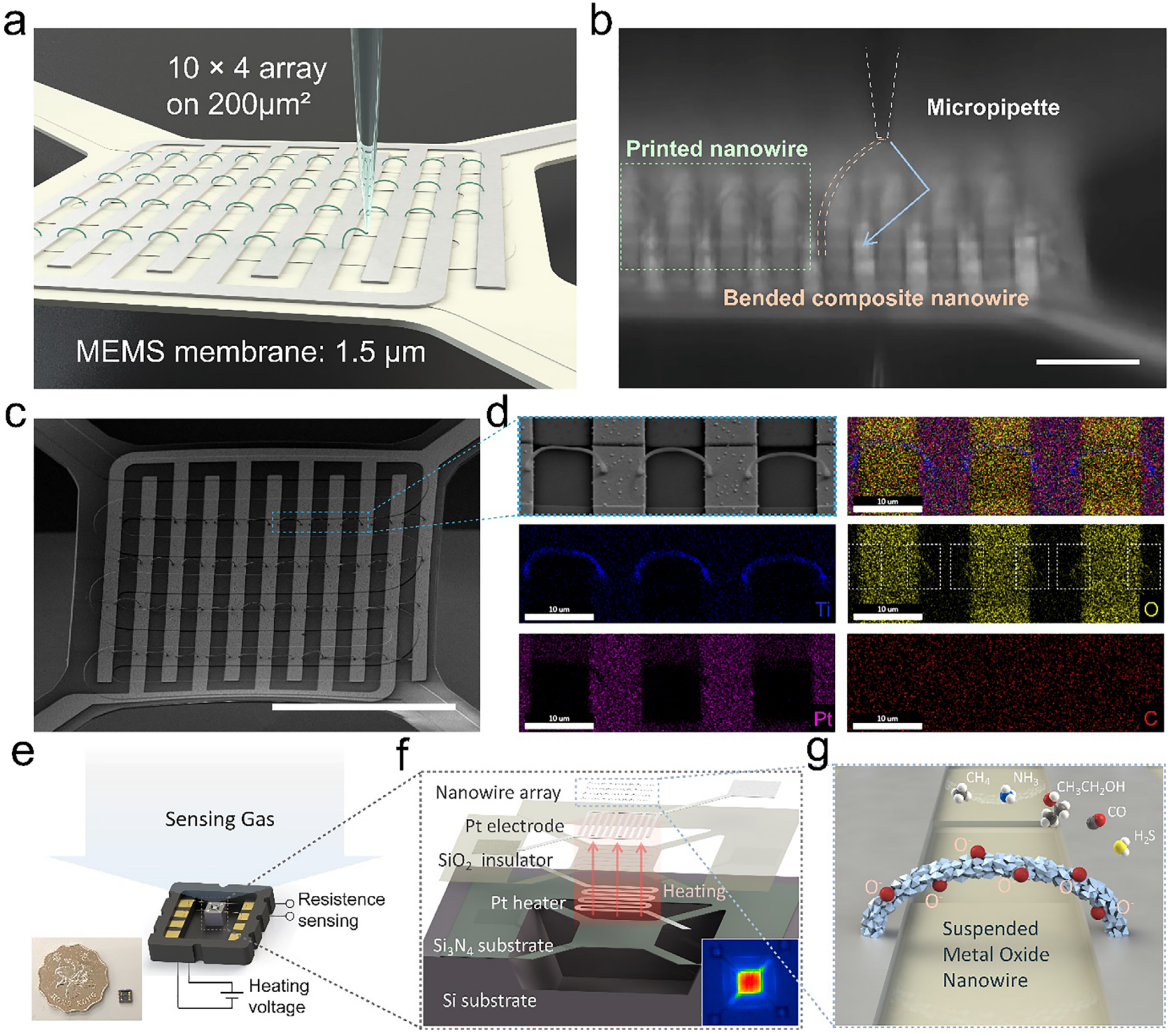
Direct printing of MOX nanowires array on MEMS chip as a gas sensor. a) Schematic illustrations showing the printing process of a nanowire on IDEs and b) corresponding real‐time optical micrograph. c) FE‐SEM image of the calcinated 4×10 TiO_2_ nanowires array on MEMS microheater membrane. d) Magnified FE‐SEM and energy‐dispersive X‐ray spectroscopy (EDS) images of three TiO_2_ nanowires printed on Pt IDE after calcination. e) Schematic diagram illustrating the sensing circuit and (inset) size comparison between a gas sensing MEMS chip and a two‐dollar coin of Hong Kong. f) Schematic diagram illustrating the exploded view of the sensor chip consisting of Si substrate, Si_3_N_4_ suspended substrate, Pt heater, SiO_2_ insulator, Pt IDEs, 3D printed MOX nanowire array (From bottom to top). Inset: corresponding IR image in heating. g) The gas sensing mechanism is made by polycrystalline MOX nanowire. Scale bars: b) 30 µm; c) 100 µm; d)10µm.

By substituting conventional substrates with MEMS microheaters, we accomplished the in situ fabrication of MOX nanowire arrays directly on the MEMS membrane. **Figure** **3**a presents the corresponding schematic illustration of this process, while Figure 3b displays a real‐time monochrome microscopic image. The printed suspended nanowire arch is visible within the green dashed box on the left. Both termini of the nanowire demonstrate the precise connection to the bright (high reflectivity) Pt IDEs. Through controlled moving path (blue arrow) of the micropipette (white dashed lines), the MOX‐PVP composite nanowire (orange dashed line) being printed undergoes controlled bending, enabling its opposite end to connect accurately to fresh electrodes ‐ a process that effectively demonstrates the spatial precision of our nanowire printing technique at the microscale.

Following the calcination procedures demonstrated in Figure 1, the FE‐SEM image of the printed TiO_2_ nanowire arrays on the MEMS membrane is presented in Figure 3c, with an enlarged view of three representative arrayed nanowires shown within the blue dashed box in Figure 3d. The nanowire arrays presented here were calcined at 600 °C. This temperature is critical as it ensures the complete removal of the sacrificial organic PVP, leading to a high‐purity metal oxide crystalline phase that is essential for achieving optimal gas‐sensing performance. To truly make our 3D printing approach CMOS‐compatible, further study to reduce calcination temperature would be necessary. A possible approach is to utilize lower‐molecular‐weight PVP, confirmed by our preliminary result in Figure S4 (Supporting Information), where the calcination temperature is reduced to 500 °C. Comprehensive chemical analysis was performed by energy‐dispersive X‐ray spectroscopy (EDS), as evidenced in Figure 3d. The EDS elemental mapping confirms homogeneous Ti distribution throughout the nanowire structure. Oxygen distribution (yellow) clearly delineates both the SiO_2_ insulating layer and TiO_2_ nanowires (termini marked by white dashed boxes), while platinum (purple) mapping corresponds to the Pt IDEs. The uniform carbon distribution profile conclusively demonstrates negligible polymeric residue within the nanowires.

The MOX nanowire arrays were directly integrated on a MEMS chip, demonstrating their gas‐sensing capabilities. Figure 3e–g illustrates the configuration of the MEMS microheater‐based gas sensor, detailing its structural design and operational principle. The chip is configured with two pairs of electrodes, one for chemiresistive gas sensing and the other for Joule heating of the sensor, encapsulated in an Al_2_O_3_ ceramic package. Even with the package shell, the overall size of the chip is far smaller than a two‐dollar coin in Hong Kong (the inserted photo of Figure 3e). The explosive view of the gas sensor chip is illustrated in Figure 3f, illustrating the sequential stacking of components: a Si substrate with a microcavity at the center, a suspended Si_3_N_4_ membrane holding the sensing part, a layer of Pt serpentine resistor as a heating unit, a SiO_2_ insulator, Pt IDEs, and an array of MOX nanowires as a sensing material. This air‐floating configuration reduces excessive heat dissipation through the bulk Si substrate, leading to localized, effective heating of metal oxide nanowires for gas sensing. The inset of Figure 3f shows an infrared (IR) microscope image of the fabricated chip when heating. The detailed fabrication process of the MEMS chip is described in Figure S8 (Supporting Information).

MOX‐based gas sensors typically operate at high temperatures from 100 to 450 °C. Thus, maintaining the working temperature is costly in power consumption. A suspended membrane microheater has a significantly lower specific heat capacity and smaller radiant area than traditional heaters, reducing low power consumption in gas sensing. Figure S9a (Supporting Information) depicts the exploded view of the suspended membrane consisting of Pt IDEs, SiO_2_ insulating layer, and Pt micro‐heater on the Si_3_N_4_ substrate. The performance of micro‐heating was investigated by infrared imaging. Figure S9b (Supporting Information) shows a 2D temperature field produced by the micro‐heater with an applied voltage of 1 V. The temperature of a microscale sensing region reaches 110 °C, while the surroundings remain at 30 °C. The plot in Figure S9c (Supporting Information) shows the dependence of the temperature at the heater center on the applied voltage ranging from 0 to 3.0 V, and the inset shows an immediate temperature response on a stepwise temperature increase from 0.9, 1.0, to 1.1 V. The cyclic temperature variation from 25 to 355 °C was generated by sweeping the voltage between 0 and 2.5 V (Figure S9d, Supporting Information), demonstrating the reliable performance of the heater. The transverse temperature distribution plotted in Figure S9e (Supporting Information) exhibits a microscale localized heating as the pixel resolution is 15 µm, beneficial for realizing low‐power consumption, miniaturized gas sensing devices. The temperature of the suspended nanowire was estimated as only ≈1 °C lower than that of the heater, as numerically simulated in Figure S10 (Supporting Information). Such a small difference in temperature is not expected to cause significant variation in sensing performance.

The mechanical robustness of the printed nanowire sensors was characterized by a series of drop tests at different heights up to 1 m, insufficient to fracture the MEMS membrane (Figure S11, Supporting Information). Even after an extreme impact sufficient to fracture the MEMS membrane, the printed nanowires remained structurally sound, retaining their original arch structure.

The gas sensing mechanism is briefly illustrated in Figure 3g. The surface adsorption of oxygen molecules on a metal oxide nanowire leads to an equilibrium of surface chemisorbed oxygen reactions. Target gases with oxidizing or reducing properties can shift this equilibrium, causing a change in the depletion layer thickness and carrier concentration of the nanowire. This changes the electrical resistance, quantified by the resistance ratio between *R* and *R*_0_ (*R*_0_: the initial resistance).

### 2.3 Performance of MOX Nanowire‐Based Gas Sensor

**Figure 4:**
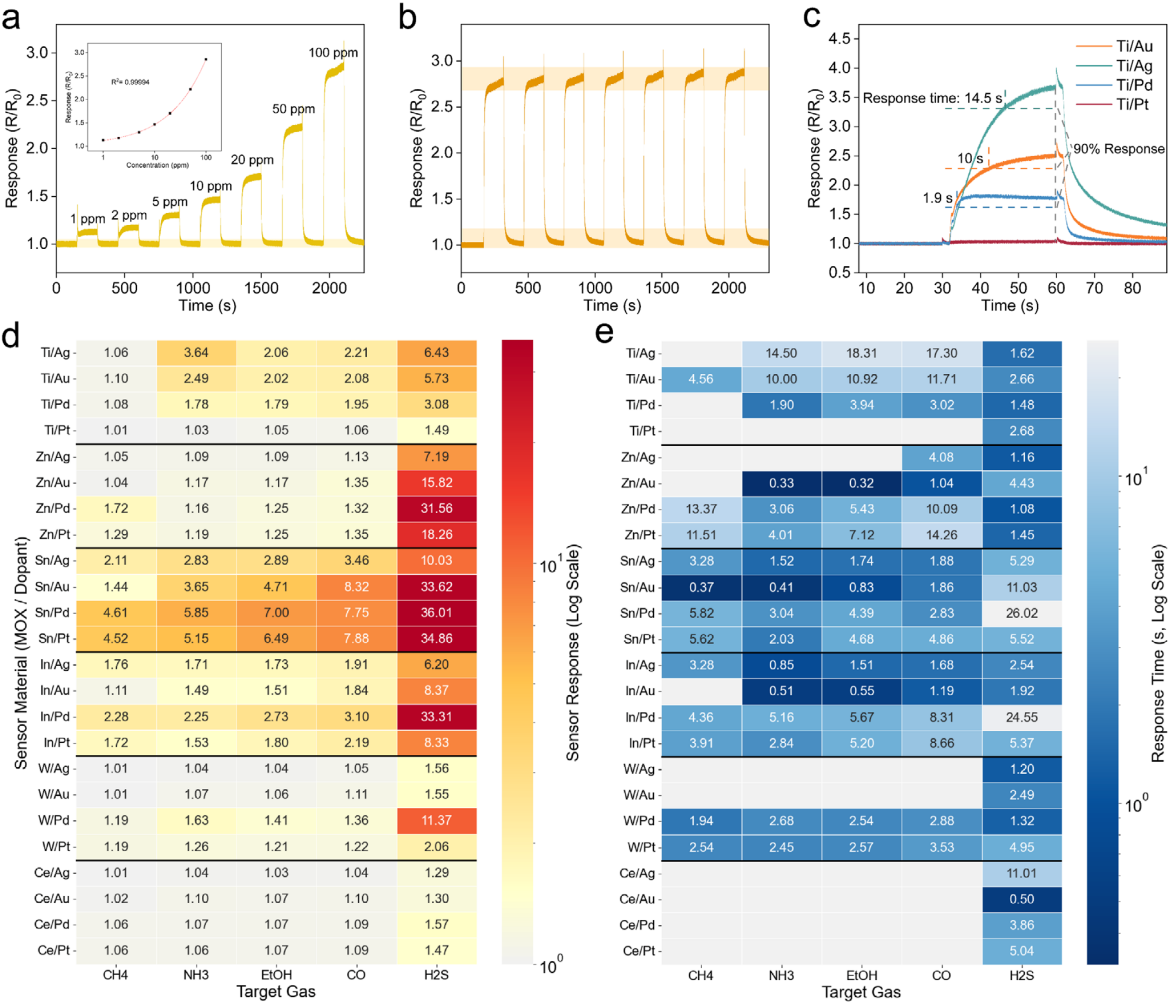
Performance of MOX nanowire‐based gas sensor. a) Real‐time chemiresistive response‐recovery curve of the Ti/Au nanowire sensor to NH_3_ gas with different concentrations from 1 to 100 ppm (applied heating voltage: 2.5 V). (Inset: plot of response—NH_3_ concentration. b) Repeating response‐recovery cycle at a constant NH_3_ concentration of 100 ppm NH_3_ (period: 300 s). c) Fine analysis of the responses of TiO_2_ nanowire sensors doped with Au, Ag, Pt, Pt to 100 ppm NH_3_ gas. d,e) Heatmap summary of the 24‐sensor array performance against five target gases (100 ppm). Both panels group the sensors by their base MOX material (Y‐axis) and use a logarithmic color scale. d) Heatmap of the sensor response values, where the warm colormap transitions from gray (low response) to deep red (high response). e) Heatmap of the sensor response times (s), where the cool colormap transitions from deep indigo blue (fast response) to gray (slow response). Gray cells in (e) denote an insufficient response for time calculation.

The gas sensing performance of MOX nanowires directly printed on the suspended membrane was quantitatively investigated. A structural feature of freestanding nanowires suspended between electrodes can offer an improvement in gas‐sensing performance. Owing to a greater specific surface area and more reliable electrical contact, freestanding nanowires can exhibit a larger gas‐sensing response and a higher signal‐to‐noise ratio compared to nanowires lying on a substrate, as quantitatively studied in Figure S12 (Supporting Information). With the superiority of the freestanding structure established, we focused our investigation on the gas‐sensing performance of freestanding TiO_2_ nanowires. To improve the performance of the sensor (response speed and selectivity), nanowires are doped with noble metals. The doping is readily done by adding noble metal precursors into the printing ink with a concentration of 1 at%. In this study, we printed MOX nanowires doped with Au (metal/Au), Ag (metal/Ag), Pd (metal/Pd), and Pt (metal/Pt), respectively, for sensing various gases. To ensure a fair comparison of intrinsic material properties, the optimal heating voltage was empirically determined to be 2.5 V. This value was selected as the standard operating condition because it yielded the highest response to 100 ppm of NH_3_ for the majority of the representative sensors tested (Ti/Ag, Ti/Pd, and Ti/Pt), as detailed in Figure S13 (Supporting Information). **Figure** **4**a shows a chemiresistive response of the printed Ti/Au nanowire sensor to NH_3_ gas with the heating voltage set at 2.5V. It is clearly shown that the amplitude of the resistive response increases as NH_3_ concentration increases from 1 to 100 ppm. This concentration dependency corresponds to a log fit, as shown in the inset of Figure 4a (*R* ^2^ = 0.9999). Figure 4b shows 7 cycles of the resistive response at 100 ppm of NH_3_, exhibiting an amplitude of 2.84 with a standard deviation of 0.036.

Figure 4c plots the response‐recovery curves of different doping metals, including Au, Ag, Pd, and Pt, for 100 ppm of NH_3_ gas. The NH_3_ sensing performance was varied with the catalytic properties of doping species. (Two small spikes at the beginning and end of the curve may result from brief interruptions in the airflow by valve switching.) The Ti/Ag nanowire sensor has the highest response of 3.67, but the longest response time (defined as the time required to reach 90% response) of ≈14.5 s. The Ti/Au nanowire sensor has a medium response of 2.51 and a response time of ≈10 s. The Ti/Pd nanowire sensor has the lowest response of 1.82 but the shortest response time of ≈1.9 s. The Ti/Pt nanowire sensor showed almost no response to NH_3_. All the sensors present a short response time compared to other fast‐response MEMS‐based sensors due to their ultrasmall sensing area resulting from the nanowire dimension, leading to a rapid equilibrium of the surface chemisorption.^[^ ^73^, ^74^, ^75^ ^]^

The integration of the nanowires in parallel aids gas‐sensing performance not only by averaging out wire‐to‐wire variation but also by reducing the device resistance. Quantitative comparison data are briefly illustrated in Figure S14 (Supporting Information). The response amplitude and time primarily depend on the material composition of the nanowire.

Not only can the response to a single gas type be changed by doping with different precious metals, but our 3D printing scheme can further be generalized for a wide range of metal oxide and noble metal compositions, potentially paving the way for manufacturing multimodal gas microsensors. A total of 24 compositions of the printed nanowire arrays were demonstrated by combining five different metal oxides of TiO_2_, ZnO, SnO_2_, In_2_O_3_, WO_3_, CeO_2_, and four noble metal dopants of Au, Ag, Pd, and Pt. Their gas performances for five gas species of CH_4_, NH_3_, CH_3_CH_2_OH, CO, and H_2_S, gases were quantitatively investigated. It is noteworthy that a simple change of precursors in the ink preparation enabled the printing of such diverse materials.

The chemiresistive responses of 24 different metal oxide nanowires to five gas species were systematically evaluated at a fixed concentration of 100 ppm. The response‐recovery curves are presented in Figure S15 (Supporting Information), and the corresponding response amplitudes and response times are summarized as heatmaps in Figure 4d,e, respectively.

From the perspective of gas species, H_2_S consistently produced the strongest responses across most sensor types. This is attributed to its strong chemical affinity for metal oxide surfaces, where it chemisorbs strongly to the surface, and in some cases even forms covalent bonds with surface atoms, significantly disturbing the surface equilibrium state.^[^ ^76^, ^77^, ^78^ ^]^ In contrast, CH_4_ exhibited the lowest response among the five gases. Due to its high thermodynamic stability and weak polarity, CH_4_ sensing typically requires catalytic activation, which explains the enhanced response upon the incorporation of highly active catalytic dopants such as Pd and Pt.^[^ ^79^, ^80^ ^]^

Considering the metal oxides, SnO_2_‐based sensors showed the highest responses, followed by those based on In_2_O_3_, TiO_2_, and ZnO. This trend can be attributed to their favorable electronic properties—specifically, high electron mobility and suitable band structures—which promote efficient charge carrier transport and the formation of a highly responsive electron depletion layer.^[^ ^81^ ^]^ For instance, SnO_2_ has an electron mobility of ≈100 cm^2^ V^−1^ s^−1^, significantly higher than the others.

In terms of doping elements, Pd‐doped sensors exhibited the highest responses to H_2_S. This is due to Pd's strong affinity toward H_2_S, which can dissociate on the surface into atomic hydrogen and sulfur.^[^ ^82^ ^]^ The latter strongly binds with Pd to form Pd–S bonds, which may lead to the formation of surface‐bound sulfur species.^[^ ^83^ ^]^ These sulfides are themselves semiconducting and can substantially modify the surface electronic structure. Moreover, the formation of surface sulfur species (e.g., Pd–S) at the Pd/MOX interface modulates the local band alignment and increases the electron depletion layer width, thereby enhancing electronic sensitization.^[^ ^83^ ^]^ Meanwhile, Pd and Pt, both with high work functions and low oxygen adsorption energies (e.g., Pd: −0.93 eV; Pt: −0.83 eV), also promote O_2_ dissociation, leading to an increased density of reactive surface oxygen species and enhancing the chemiresistive response.^[^ ^84^, ^85^, ^86^, ^87^ ^]^

Figure 4e summarizes the response times of all the sensors for the five target gases. The sensors with responses below 1.1 were excluded from response time analysis due to insufficient signal‐to‐noise ratios, which make accurate determination impractical. SnO_2_, In_2_O_3_, and ZnO generally exhibit short response times, due to the high carrier mobility (≈30–100 cm^2^ V^−1^ s^−1^) and efficient charge exchange kinetics. On the other hand, TiO_2_ shows slow dynamics due to relatively low carrier mobility (< 0.4 cm^2^ V^−1^ s^−1^) and stable surface chemistry.^[^ ^88^, ^89^ ^]^

Regarding the doping effect, Au‐doped sensors showed the shortest time. This can be attributed to Au's ability to reduce the activation energy of surface reactions, allowing near‐instantaneous initiation of gas interactions.^[^ ^85^ ^]^ On the other hand, Pd, Pt, and Ag, which interact more strongly with gas molecules, produce higher response amplitudes. This stronger binding, while enhancing sensitivity, can hinder product desorption and thus slow the establishment of equilibrium, resulting in longer response times.

These trends illustrate the response amplitude and time that arise from a combined effect of electronic and chemical sensitization. Notably, a trade‐off often exists: materials and dopants that generate high responses tend to exhibit slower dynamics due to stronger surface interactions. For instance, the Sn/Au combination yields an exceptionally fast response of 0.37 s to CH_4_, but its response value of 1.44 is considerably lower than that of Sn/Pd, which has a slower response time of 5.82 s but a higher response value of 4.61. In this way, our universal 3D printing strategy enables direct quantitative evaluation and provides a practical benchmark for future sensor development.

Figure S16 (Supporting Information) shows the reconstructed radar charts with respect to the sensing gases. CH_4_, NH_3_, CH_3_CH_2_OH, CO, and H_2_S gases were detected by 24 different nanowire sensors. The resulting multi‐modality response radar chart patterns could give multi‐dimensional characteristics to target gases and facilitate feature extraction,^[^ ^90^ ^]^ category identification,^[^ ^91^ ^]^ and increase concentration prediction accuracy.^[^ ^92^ ^]^ Additionally, this method achieves nanowires with similar overall shapes through a universal preparation method, which can be used to evaluate the intrinsic properties of a certain material combination for a certain gas sensing from a single perspective, and can be used for material screening. A detailed performance comparison of our sensor with other recently reported MEMS‐based sensors is provided in Table S2 (Supporting Information).

### 2.4 AI‐Optimized Combinatorial Gas Fingerprinting

**Figure 5:**
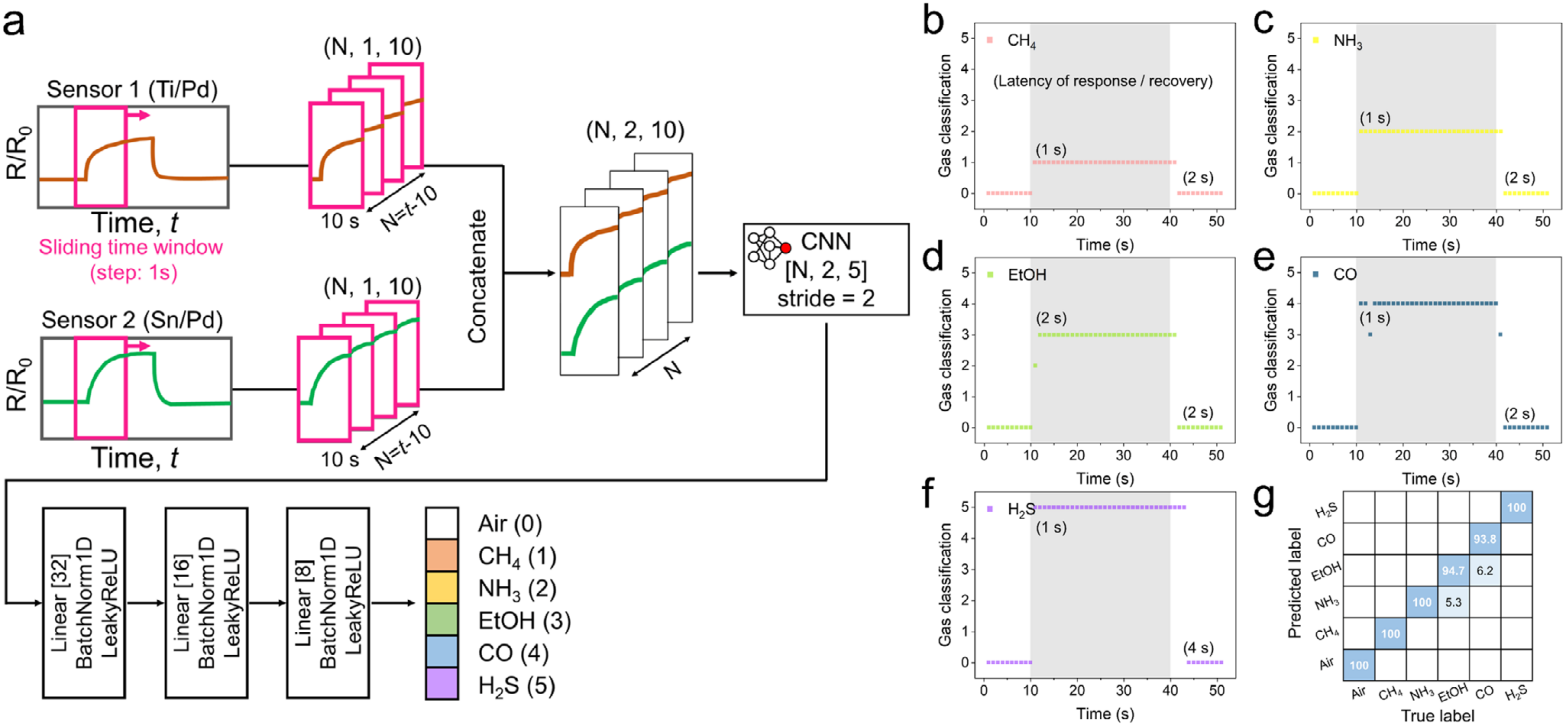
Gas‐species selectivity ML modeling. a) CNN architecture of the model used for gas classification. Data collected from Sensor 1 (Ti/Pd) and Sensor 2 (Sn/Pd) were processed using a 10 s sliding time window and concatenated into a (2,10) matrix. This matrix was then passed through CNN computation using two‐channel filters of size (2,5), followed by fully connected (FC) layers, ultimately outputting the gas label with the highest probability. b–g) Gas classification results of the CNN model developed using the Ti/Pd and Sn/Pd sensor array. The model enables real‐time classification of b) CH_4_, c) NH_3_, d) EtOH, e) CO, and f) H_2_S with high accuracy and extremely low latency. g) Summary of gas type classification results presented in the confusion matrix.

To demonstrate the scalability of our approach, we employed machine learning algorithms to validate the gas species selectivity of 3D‐printed MOX nanowire sensors.^[^ ^93^ ^]^ Specifically, we developed a CNN‐based prediction model using raw sensing data from two material systems (Ti/Pd and Sn/Pd nanowires), with the detailed network architecture illustrated in **Figure** **5**a. Given the supervised nature of CNNs, ground truth labels are provided corresponding to the temporal gas response data. The labels were assigned as follows: normal air, CH_4_, NH_3_, CH_3_CH_2_OH(EtOH), CO, and H_2_S were labeled as 0 to 5, respectively.

The experimental dataset contained three measurement cycles (shown in Figures S17 and S18, Supporting Information). Each gas exposure lasted 30 s within a 60 s measurement cycle. For each sensor, time‐series data were acquired for five different target gases across repeated measurement cycles. For model training, two cycles were allocated as training data, while the remaining cycle was randomly split into validation and test data in a 1:1 ratio. The final data distribution was set as a train: validation: test = 2: 0.5: 0.5. No additional processing, such as smoothing, was applied to the raw data. To generate input samples for the ML model, we applied a 10 s sliding time window with a stride of 1 s to the temporal gas response data (normalized as *R*/*R*_0_), capturing the unique transient behavior of each gas. This process produced 51 segments per cycle (e.g., time points 1–10, 2–11, …, 51–60), with each segment represented as a 1 × 10 matrix.

After pre‐processing, the data from the two selected sensors were concatenated into a 2 × 10 matrix as input to the model. The two sensors—Sn/Pd and Ti/Pd—were chosen based on their complementary response characteristics. Sn/Pd exhibited the strongest overall response among the various material combinations, which made it effective in distinguishing normal air from gas exposure. However, its much stronger response to H_2_S compared to other gases could potentially mask the responses to the remaining four target gases. In contrast, Ti/Pd showed relatively balanced sensitivity across all target gases, making it a suitable complementary sensor. For CNN training, the loss function was calculated using cross‐entropy, and a learning rate (*η*) of 10^−4^ was used. Two filter sizes of (2,5) were applied, with a stride of 1, and the model was trained for 400 epochs. Batch normalization and LeakyReLU were used as activation functions in all layers.

After passing through fully connected layers, the model generated six output nodes representing gas probabilities, including air, with the final classification determined by the highest probability. As seen in Figure 5b–g, there is a slight error in distinguishing EtOH and CO. This is because of their similar response patterns under 100 ppm. Nevertheless, the model is still able to differentiate them due to the application of a sliding time window, which allows it to capture transient response characteristics over time, such as differences in slope and overall response shape, as features for learning. The accuracy of this model was defined as the ratio of correctly predicted cases over the total experiment time, and the model achieved an accuracy of 98.0%.

## 3 Conclusion

In this work, we have developed a universal direct 3D nanoprinting method to fabricate metal oxide crystalline nanowires down to 180 nm in diameter and with a capability of 500 µm in length. This method can be extended to a wide range of metal oxide semiconducting materials and offers significant flexibility in material combinations. The printed nanowire arrays exhibited well‐defined shapes and polycrystalline nature, facilitating rapid equilibrium of the surface chemisorbed oxygen reaction. Fabricating a nanowire array on a suspended MEMS membrane heater results in high‐performance gas sensors with short response times (seconds‐level), combinatorial selectivity, and low power consumption. This heterogeneous integration is not easily attainable by traditional manufacturing approaches. The fabrication of diverse metal oxide nanowire‐based sensors (24 types) generates multimodal response profiles, enabling multidimensional gas fingerprinting through radar chart pattern analysis. AI‐driven combinatorial fingerprinting via convolutional neural networks, which leverages transient response dynamics to achieve 98.0% classification accuracy for five gases (CH_4_, NH_3_, CH_3_CH_2_OH, CO, and H_2_S). By unifying 3D printing, MEMS, and machine learning, our scheme could be generalized to fabricate multi‐material, heterogeneous integrated circuit electronic devices.

## 4 Experimental Section

### MEMS Microheater Fabrication

Figure S7 (Supporting Information) shows the schematic of the MEMS fabrication process. Initially, a ≈1 µm thick silicon nitride film was grown on a 4 in. silicone <100> wafer via low‐pressure chemical vapor deposition (LPCVD). Electron beam evaporation and double‐layer lift‐off processes were carried out to pattern the platinum serpentine resistor as a microheater on the wafer. Next, the wafer was coated with a ≈500 nm thick silicon oxide film using plasma‐enhanced chemical vapor deposition (PECVD), and a platinum interdigitated electrode was deposited on it using the same process as the serpentine resistor. Subsequently, the silicon nitride and silicon oxide membrane was selectively dry etched by inductively coupled plasma (ICP) etching to uncover the microheater bonding pads and the wet etching windows. The microheater and the interdigitated electrode were suspended by a silicon cavity and four beams via anisotropic wet etching with a 40 wt% KOH at 60 °C for 9.5 h. Finally, a ceramic package was installed by commercial support to simplify the electrode links and protect the chip.

### Material Preparation

The 2 wt% polyvinylpyrrolidone (PVP) solution was prepared by dissolving PVP (0.4 g. *M*w ≈ 1300 000, Sigma‐Aldrich) in dimethylformamide (DMF, 20 mL). In a typical experiment, the Ti precursor ink was prepared by dissolving titanium diisopropoxide bis(acetylacetonate) (TAA, 48.81 µL) and 0.5 mL 2 wt% PVP solution in DMF (3.5 mL) to keep the Ti molar concentration equal to 25 mmol L^−1^. Likewise, 6.25, 12.5, 50, and 100 mmol L^−1^ Ti precursor ink were prepared by changing the amount of TAA. Au, Ag, Pt, and Pd doped Ti precursor inks were prepared by adding gold (III) chloride hydrate, silver nitrate, chloroplatinic acid hexahydrate, and tetraamminepalladium (II) nitrate to the Ti precursor ink, and the atomic ratio of the doped elements was set to 1 at%. To prepare others 20 kinds of printing inks, zinc acetate dihydrate, tin (II) chloride dihydrate, indium (III) nitrate hydrate, ammonium metatungstate hydrate, and cerium (III) chloride hydrate are used as the metal oxides precursors. The molar concentration of the metal elements in the inks was set to 25 mmol L^−1^, and the dopants' atomic ratios were set to 1 at%. For low calcination temperature (500 °C) ink, 1300k PVP was replaced with 10k PVP. Prior to use, all solutions were stirred for 0.5 h at room temperature. The printing nozzle and borosilicate micropipettes with a 2 µm tip diameter were fabricated using a programmed heat‐pulling process (P‐97 Flaming/Brown Micropipette Puller, Sutter Instrument).

### 3D Printing and Calcination

The customized 3D printing system comprised an ink‐filled micropipette, a building platform, and two microscopes oriented in different directions. The building platform is controlled by a three‐axis (x, y, z) stepper motorized stage (XA05A, ZA05A, Kohzu Precision) and two manually tilted stages. A customized heating substrate is installed on the building platform to regulate the substrate temperature accurately. The printing process is monitored in real‐time using a side‐view optical microscope with a 20X long working distance objective (Mitutoyo) connected to a CMOS camera (DCC1545M, Thorlabs), and an orthographic‐view digital microscope (B011, Supereyes). The entire system is connected to a computer and operated using a customized LabVIEW program. After printing, the sample was placed on the building platform and baked at 353 K for 10 h before calcination. The pre‐baked sample was calcinated in the air at 600 °C (for 1300 k PVP ink) and 500 °C (for 10k PVP ink) for 2 h with a ramping rate of 1 °C min^−1^ to remove the solvent and organic matter, resulting in polycrystalline phase metal oxide nanowires.

### Characterizations

The morphology of the metal oxide nanowires was characterized using FE‐SEM (Merlin, ZEISS) and TEM (Talos F200X G2, Thermo Fisher). The arc length of the suspended nanowires was measured from FE‐SEM images taken at a 45° tilt angle, and the projected 2D curve length was first measured using ImageJ software; then, the true arc length was calculated by geometrically correcting for the foreshortening effect caused by the sample tilt. The TEM sample was prepared by directly printing a ≈200 µm composite wire on a nine‐window silicon TEM grid, followed by calcinating with the same calcination recipe. EDS mapping was performed using an FE‐SEM (Merlin, ZEISS) at 20 kV. The thermal response mapping of the MEMS microheater was characterized using an infrared camera (ImageIR 8300 hp, InfraTec) with a pixel size of 15 µm × 15 µm.

### Thermal Simulation of Nanowires on MEMS Microheater

Steady‐state thermal simulations of the MEMS sensor were conducted by COMSOL Multiphysics using coupled heat transfer in solids and fluids and electric currents modules. Joule heating of the platinum heater and natural convection in the surrounding air were modeled.

### Gas Sensing

Gas sensing measurements were carried out using a commercial gas sensing measurement system (JF02F) to determine the response to NH_3_, CH_3_CH_2_OH, CO, H_2_S, and CH_4_. The applied voltage for measuring the sensor resistance is 5 V. The response was calculated as the ratio between *R* and *R*_0_, and the response times were determined as the time required to reach 90% of the highest response variation when exposed to the target gas and compressed air. To investigate the optimal sensing temperature, measurements were conducted under different heating voltages from 1.9 to 2.7V. In a typical experiment, sensing was performed at a heating voltage of 2.5 V.

## Conflict of Interest

The authors declare no conflict of interest.

## Supporting information



Supporting Information

## Data Availability

Research data are not shared.
