# Supplementary material for: Universal 3D‐Printing of Suspended Metal Oxide Nanowire Arrays on MEMS for AI‐Optimized Combinatorial Gas Fingerprinting
Source: Adv Sci (Weinh). 2025 Aug 26;12(43):e11794. doi: 10.1002/advs.202511794 (PMC12631845; doi:10.1002/advs.202511794)
Supplement: Supplementary file 1 — Supporting Information [file ADVS-12-e11794-s001.docx]

Supporting Information

Universal 3D-Printed Suspended Metal Oxide Nanowire Arrays on MEMS for AI-Optimized Combinatorial Gas Fingerprinting

Yu Liu^1,2^, Kichul Lee^3^, Hongjun Liu^1^,Chenwei Li^1^, Xiaoyi Zeng^1^, Rongyue Liu^1^, Yulong Chen^4^, Zijun Chen^1^, Jihyuk Yang^2^, Xiao Huan^2^, Inkyu Park^3,*^, Ji Tae Kim^2,3,*^, Xing Cheng^1,5,*^

Y. L., H. L., X. Z., Z. C., X. C.

^1^The Greater Bay Area University Joint Laboratory of Micro- and Nanofabrication, Department of Materials Science and Engineering, Southern University of Science and Technology, Shenzhen 518055, China

E-mail: [chengx@sustech.edu.cn](mailto:chengx@sustech.edu.cn)

Y. L., J. Y., X. H., J. T. K.

^2^Department of Mechanical Engineering, The University of Hong Kong, Pokfulam Road, Hong Kong, 999077, China

Email: jitae.kim@kaist.ac.kr

K. L., I. P., J. T. K.

^3^Department of Mechanical Engineering, Korea Advanced Institute of Science and Technology (KAIST), Daejeon 34141, Republic of Korea

Email: inkyu@kaist.ac.kr, jitae.kim@kaist.ac.kr

Y. C.

^4^Industrialization Center of Micro & Nano ICs and Devices, Sino-German College of Intelligent Manufacturing, Shenzhen Technology University, Shenzhen 518118, China

X. C.

^5^Advanced Materials Innovation Center, Jiaxing Research Institute of Southern University of Science and Technology, Jiaxing 314031, China

**Table S1.** Recent reports in 3D micro-/nanoprinting for fabricating metal oxide nanostructures.

| Paper reference | | Principle | Build environment | Substrate | Materials | Doping materials | Min Diameter [nm] |
| --- | --- | --- | --- | --- | --- | --- | --- |
| This work | meniscus-guided 3D printing | | air | MEMS membrane/TEM grids/silicon wafer | TiO_2_ / SnO_2_ / In_2_O_3_ / ZnO / CeO_2_ / WO_3_ | Au/Ag/Pd/Pt | 180 |
| ^[1]^ | direct ink writing | | air | silicon wafer with sacrificial layer | ZnO / CuO / In_2_O_3_ / Ga_2_O_3_ / Y_2_O_3_ / TiO_2_ / BaTiO_3_ / SrTiO_3_ | / | 766 |
| ^[2]^ | direct ink writing | | air | MEMS membrane/silicon wafer | SnO_2_ | / | 2000 |
| ^[3]^ | electrohydrodynamic | | air | silicon wafer | ZnO / SnO / In_2_O_3_ / WO_3_ / IZO | In/Ga/MO NPs | 57 |
| ^[4]^ | multi-photon lithography | | liquid | glass/quartz | ZnO / CuO / ZrO_2_ | Al | 35 |
| ^[5]^ | two-photon polymerization | | liquid | glass | Cr_2_O_3_ / MnO_2_ / ZnO / Co_3_O_4_ / NiO / Al_2_O_3_ / MgO | / | 391 |

**Table S2.** Comparison of Performance Metrics for Recently Reported MEMS-based Gas Sensors.

| Sensing Material | Morphology | Dopant | Operating Temp. [°C] | Target Gas | Response | Response time [s] | Reference |
| --- | --- | --- | --- | --- | --- | --- | --- |
| ZnO | Nanoflake | CuO | 300 | Acetone / 10 ppm | 2.264 | 22 | ^[6]^ |
| ZnO | Film | TeO_2_ | 200 | NO_2_ / 1 ppm | 1.14 | 13 | ^[7]^ |
| ZnO | Film | Ni/Cu/Co/Au/W | 37.5 | Ethanol / 100 ppm | 20-45 | ~ 80 | ^[8]^ |
| TiO_2_ | Core-shell nanowire | SnO_2_ | 300 | H_2_S / 5ppm | 5.9 | 25 | ^[9]^ |
| TiO_2_ | Film | / | RT | NO / 8 ppm | 1.2 | 109 | ^[10]^ |
| SnO_2_ | Nanoparticle | Pd | 400 | Ethanol / 10 ppm | 25 | 17.6 | ^[11]^ |
| SnO_2_ | Nanoparticle | Pd-Au/Ni/Cu | / | H_2_S / 1 ppm | 2-14.2 | 66 | ^[12]^ |
| SnO_2_ | Nanoparticle | Pt | 400 | Ethanol / 100 ppm | 2.5 | 4 | ^[13]^ |
| SnO_2_ | Nanotube | ZnO | 280 | H_2_S / 1ppm | 2.06 | 93 | ^[14]^ |
| SnO_2_ | Nanocomposite | Pd/In_2_O_3_ | 272 | Acetone / 10 ppm | 12.4 | 4 | ^[15]^ |
| In_2_O_3_ | Nanosphere | Ga | 180 | HCHO / 100 ppm | 110.6 | 5.2 | ^[16]^ |
| In_2_O_3_ | Nanofiber | Au | 277 | HCHO / 5 ppm | 3.87 | 109 | ^[17]^ |
| WO_3_ | Nanoparticle | / | 142 | NH_3_ / 1.3ppm | 1.157 | 59 | ^[18]^ |
| CeO_2_ | Nanoflower | Ni | 200 | H_2_S / 500 ppb | 3.1 | 8 | ^[19]^ |
| CeO_2_ | Nanosphere | W | 225 | Ethanol /10 ppm | 10 | / | ^[20]^ |
|  |  |  |  |  |  |  |  |
| TiO_2_ | Nanowire | Ag | 350 | H_2_S / 100 ppm | 6.43 | 1.62 | This Work |
| ZnO | Nanowire | Pd | 350 | H_2_S / 100 ppm | 31.56 | 1.08 | This Work |
| SnO_2_ | Nanowire | Pt | 350 | H_2_S / 100 ppm | 34.86 | 5.22 | This Work |
| In_2_O_3_ | Nanowire | Au | 350 | H_2_S / 100 ppm | 8.37 | 1.92 | This Work |
| WO_3_ | Nanowire | Pd | 350 | H_2_S / 100 ppm | 11.37 | 1.32 | This Work |
| CeO_2_ | Nanowire | Au | 350 | H_2_S / 100 ppm | 1.3 | 0.5 | This Work |


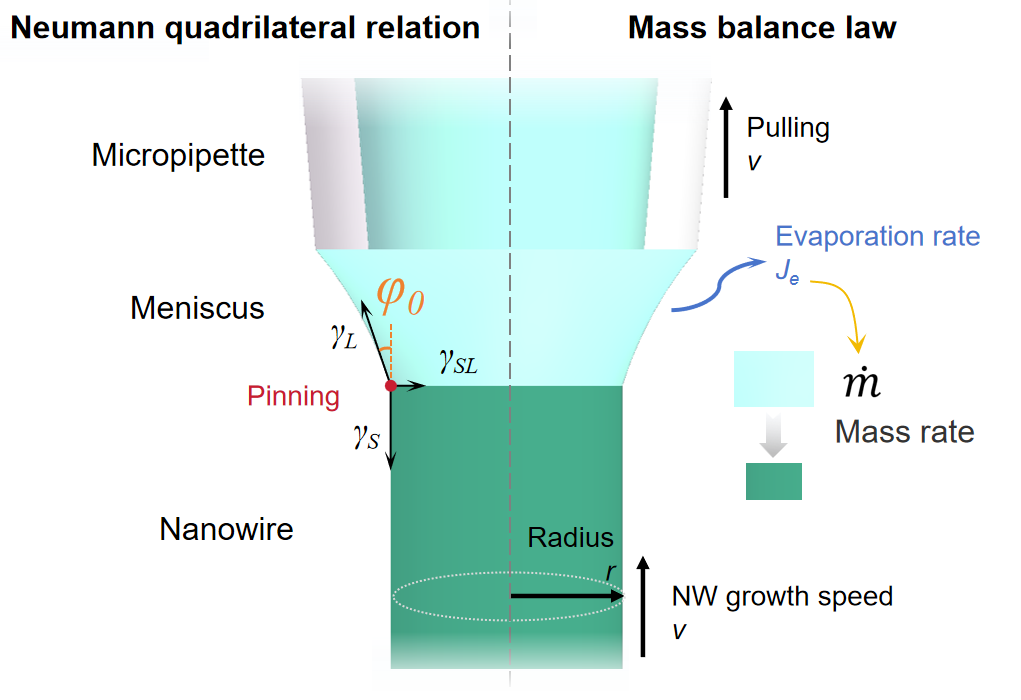


**Figure S1.** **Meniscus-guided printing mechanism. Left: Neumann quadrilateral relation: right Mass balance law.** The equilibrium stability of the meniscus is governed by the classical Neumann quadrilateral relation, which describes the vectorial force balance among interfacial tensions at the three-phase boundary,

$$\varphi_{0}=arccos\left( \frac{\gamma_{L}^{2}+\gamma_{S}^{2}-\gamma_{SL}^{2}}{2\gamma_{L}\gamma_{S}} \right)$$

where φ_0_ is defined as the angle between the growth direction and the slope of the meniscus at the three-phase contact line, γ_L_, and γ_S_ are the surface energies of the ink and the grown solid, respectively, and γ_LS_ is the interfacial energy of the ink–solid interface. This relationship defines the equilibrium angle φ_0_ at the meniscus–nanowire boundary. During the printing process, the contact angle φ can deviate within a limited adjustable range around its equilibrium value φ_0_. The bound is constrained by the meniscus stability limit governed by Rayleigh–Plateau instability and the pinning strength at the three-phase contact line. This limitation is evidenced by the formation of uneven nanowires in the unstable printing regime observed in Figure 1c. Second, the printed nanowire diameter is governed by the mass balance between the solidification rate and the pulling speed，

$$\dot{m}=\rho\pi r^{2}v$$

where $\dot{m}$ is the mass rate of solute precipitation governed by the solvent evaporation rate, and *ρ* is the density of the grown solid structure, *r* is the printing wire radius, and *v* is the nanowire growth speed, which follows the pipette pulling speed. Together, these two criteria constrain the meniscus stability and ensure the continuity of nanowire growth. When the actual angle φ remains within the allowable deviation, the meniscus remains stable, enabling repeatable and uniform printing.

**
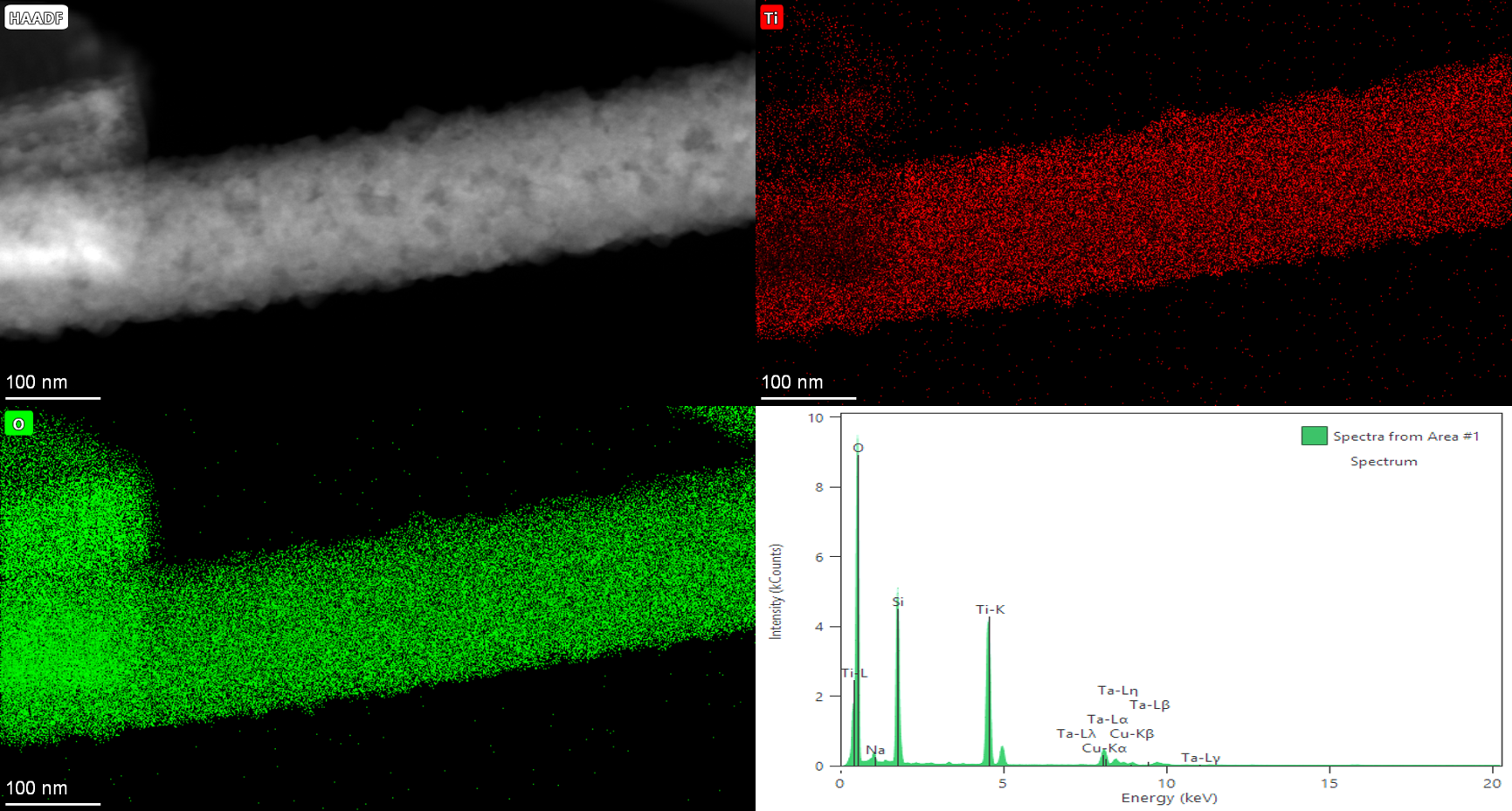
**

**Figure S2.** TEM image and EDS of the 180 nm diameter TiO_2_ nanowire.


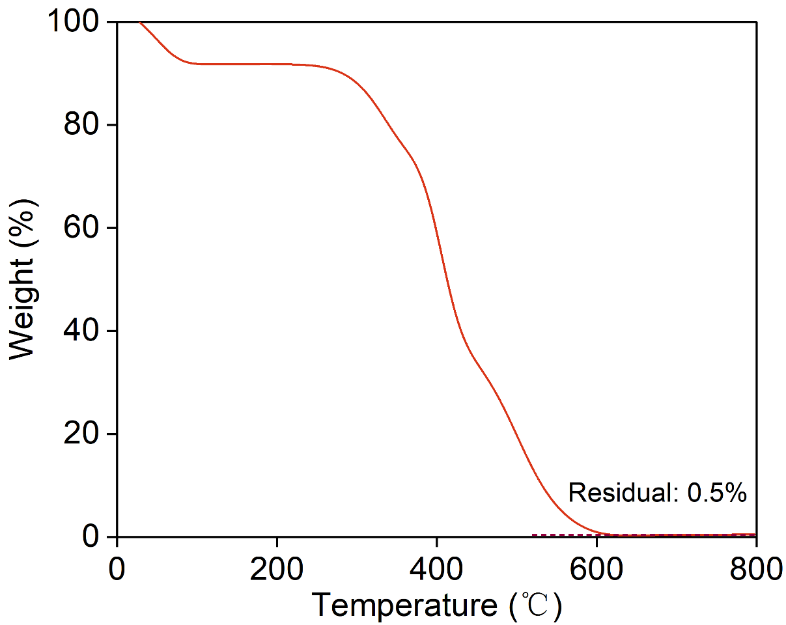


**Figure S3.** Thermal gravimetric analysis (TGA) measurements of PVP.


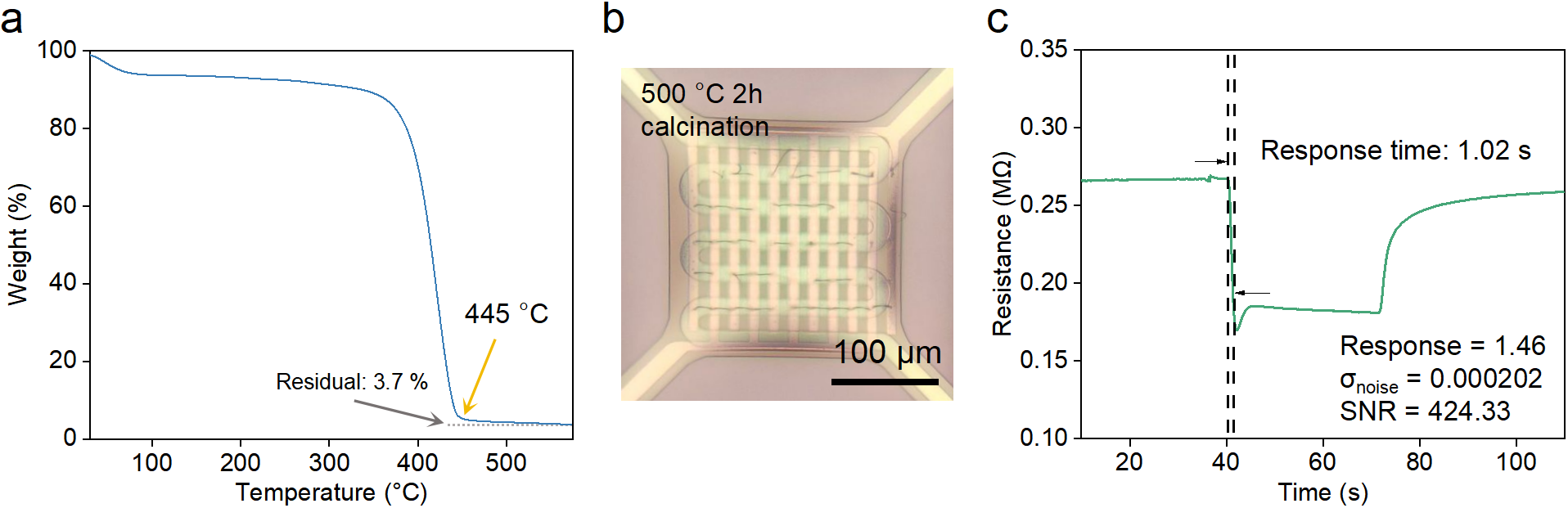


**Figure S4.** SnO_2_/Pd nanowire sensor prepared with a lower molecular weight PVP. (a) TGA curve of the low molecular weight (10k) PVP precursor in a nitrogen atmosphere, indicating that the PVP mostly decomposes below 500°C. (b) Optical micrograph of the SnO_2_/Pd nanowire array successfully fabricated on a MEMS device after calcination at 500°C. (c) Real-time chemiresistive response of the 500°C-calcined sensor to 10 ppm ethanol, demonstrating a similar response value of 1.46.


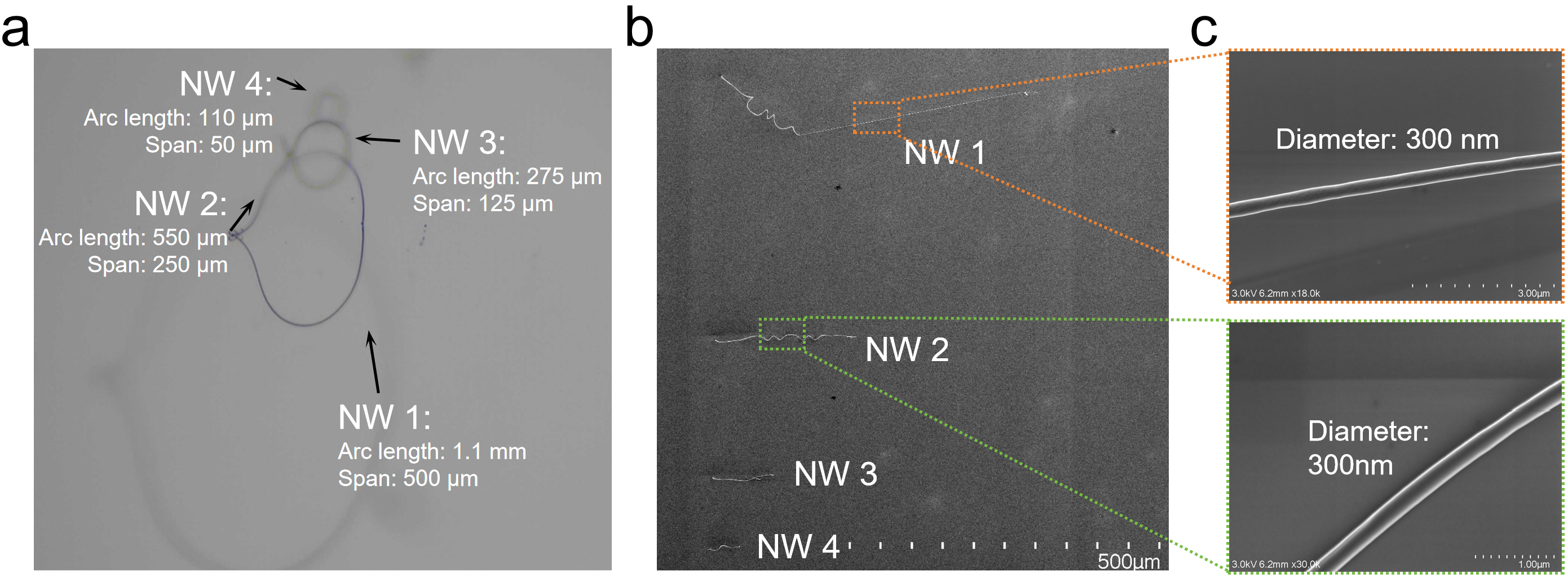


**Figure S5.** Optical and SEM images of printed nanowires on ITO substrates with lengths ranging from 110 µm to 1.1 mm, demonstrating the structural limitations of the printing process.


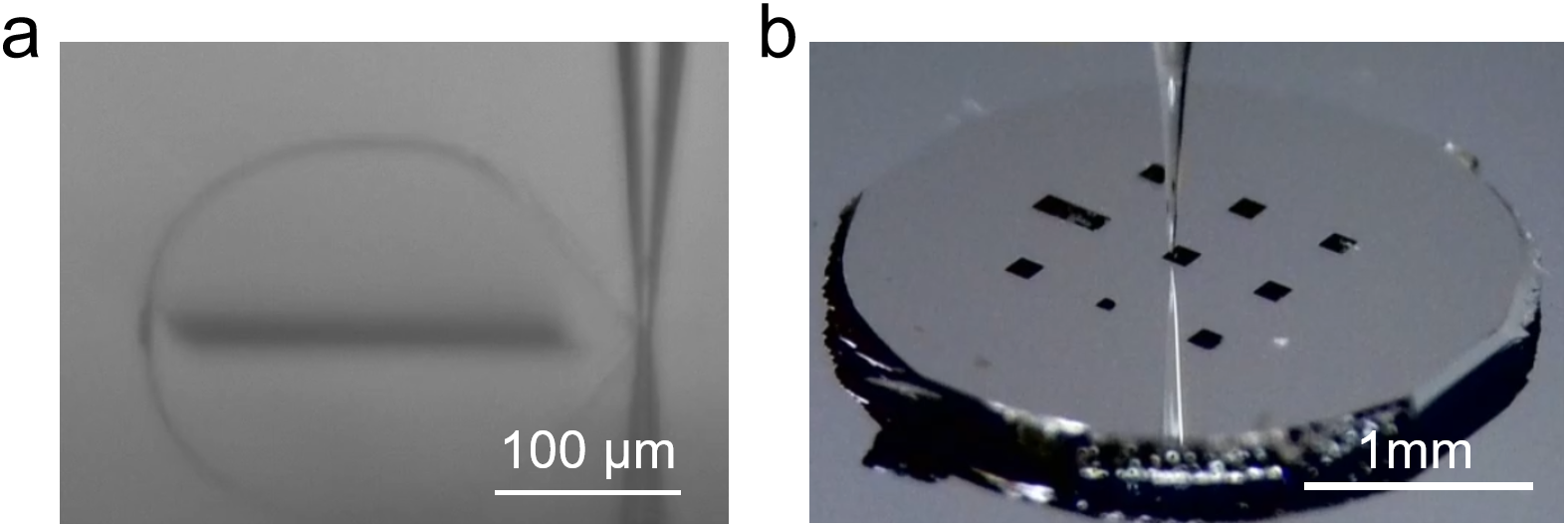


**Figure S6.** In situ fabrication of ~500 μm arc length composite nanowire on TEM grid. a) Real-time Microscope image of the printing process. b) 3D printing on Si TEM grid.


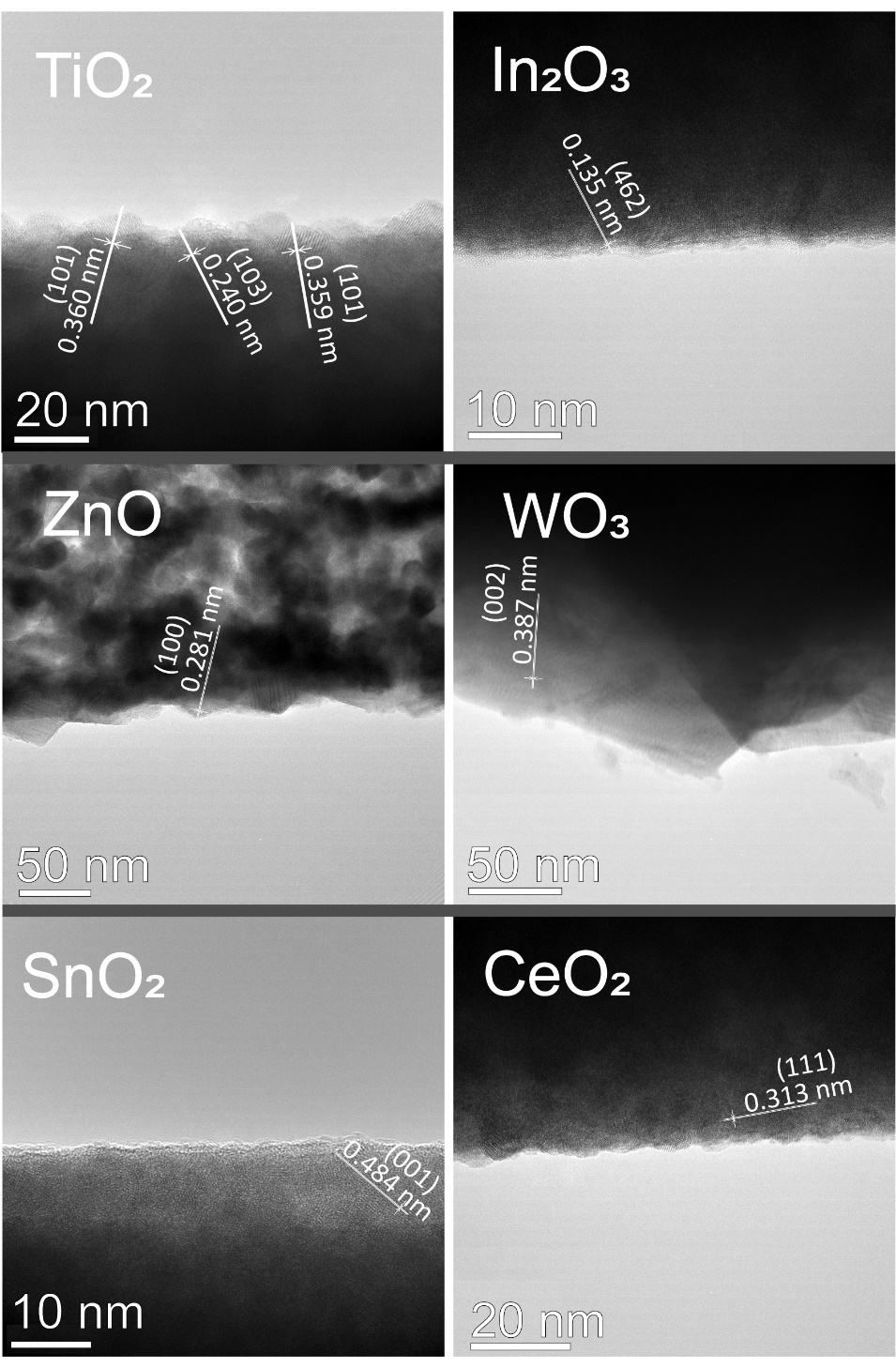


Figure S7. High-resolution transmission electron microscopy (HRTEM) images showing the edges and lattice structures of six different metal oxide nanowires, with corresponding lattice fringes labeled.


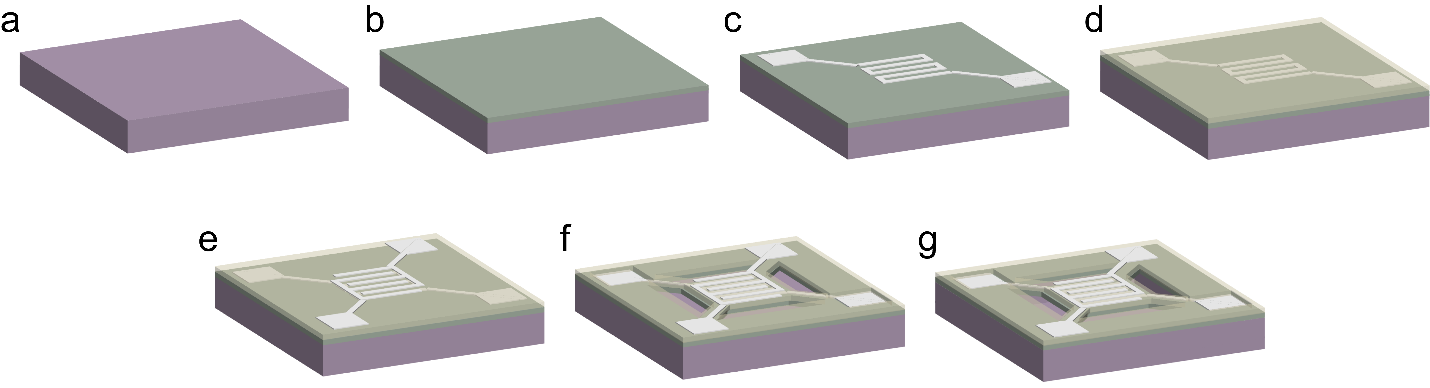


**Figure S8.** Fabrication process of the MEMS chip. a) An n-type ⟨100⟩ silicon substrate. b). A thin layer of silicon nitride is deposited by LPCVD, which provides thermal isolation between the microheater and the silicon substrate. c) The serpentine platinum resistor and the bonding pads were fabricated by double-resist lift-off processes. d) The silicon oxide insulating layer coated by PECVD. e) The IDE and the bonding pads were fabricated by double-resist lift-off processes. f) The bonding pads and the wet etching windows were uncovered through photolithography and dry etching. g) The microheater and IDE structure were suspended by anisotropic wet etching.

**
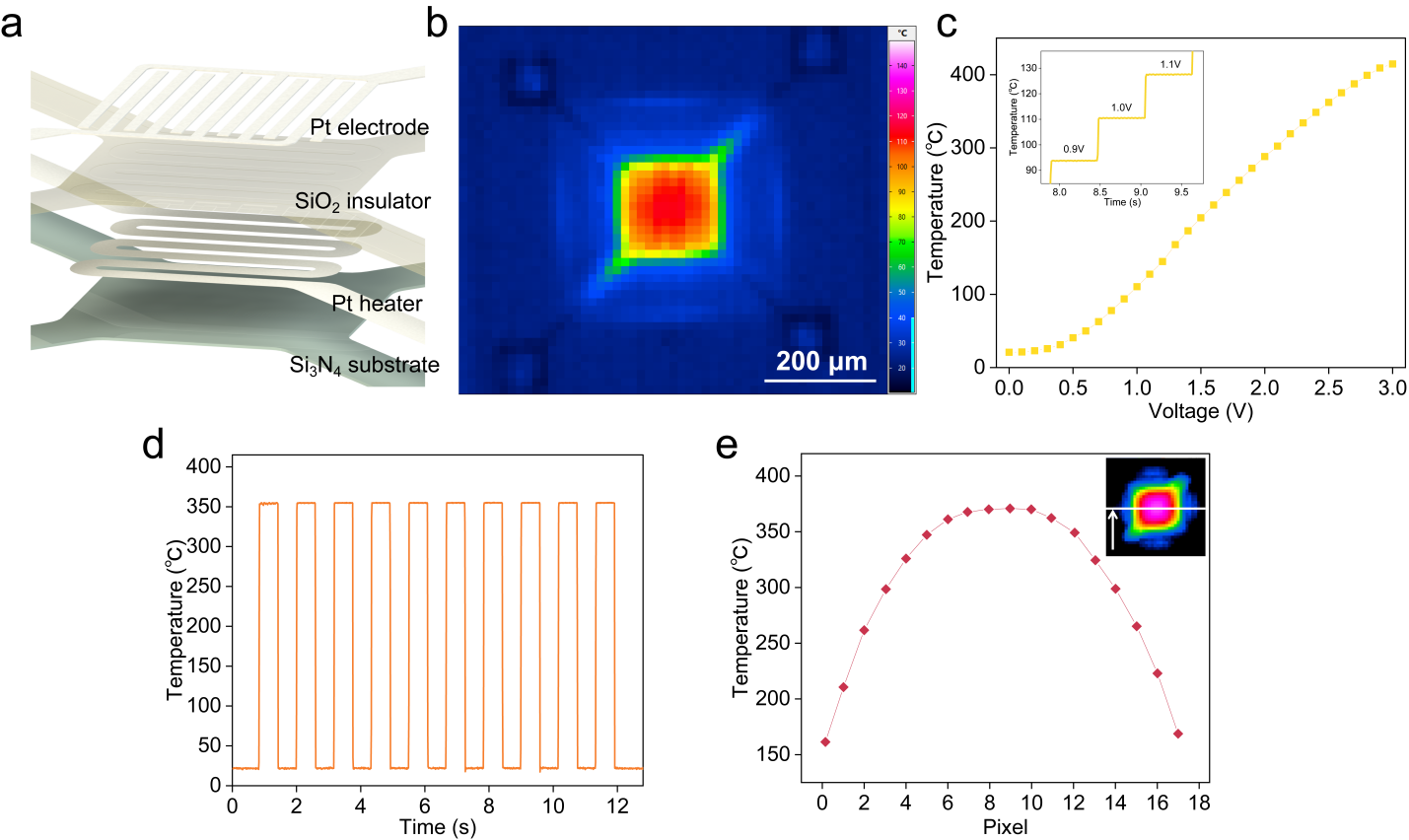
**

**Figure S9.** Performance of suspended µ-heater. a) Schematic diagram illustrating the exploded view of the suspended microheater. b) Infrared micro-image of the suspended micro heater at an applied heating voltage of 1 V (scale bar: 200 µm). c) Microheater temperature as a function of heating voltage. Inset: Stepwise temperature increase by increasing heating voltage. d) Periodic heating cycles by switching the heating voltage between 0 and 2.5 V, and the switching frequency is set as 0.85 Hz. e) Lateral temperature distribution across the microheater generated at a heating voltage of 2.5 V. (Inset: infrared micro-image).


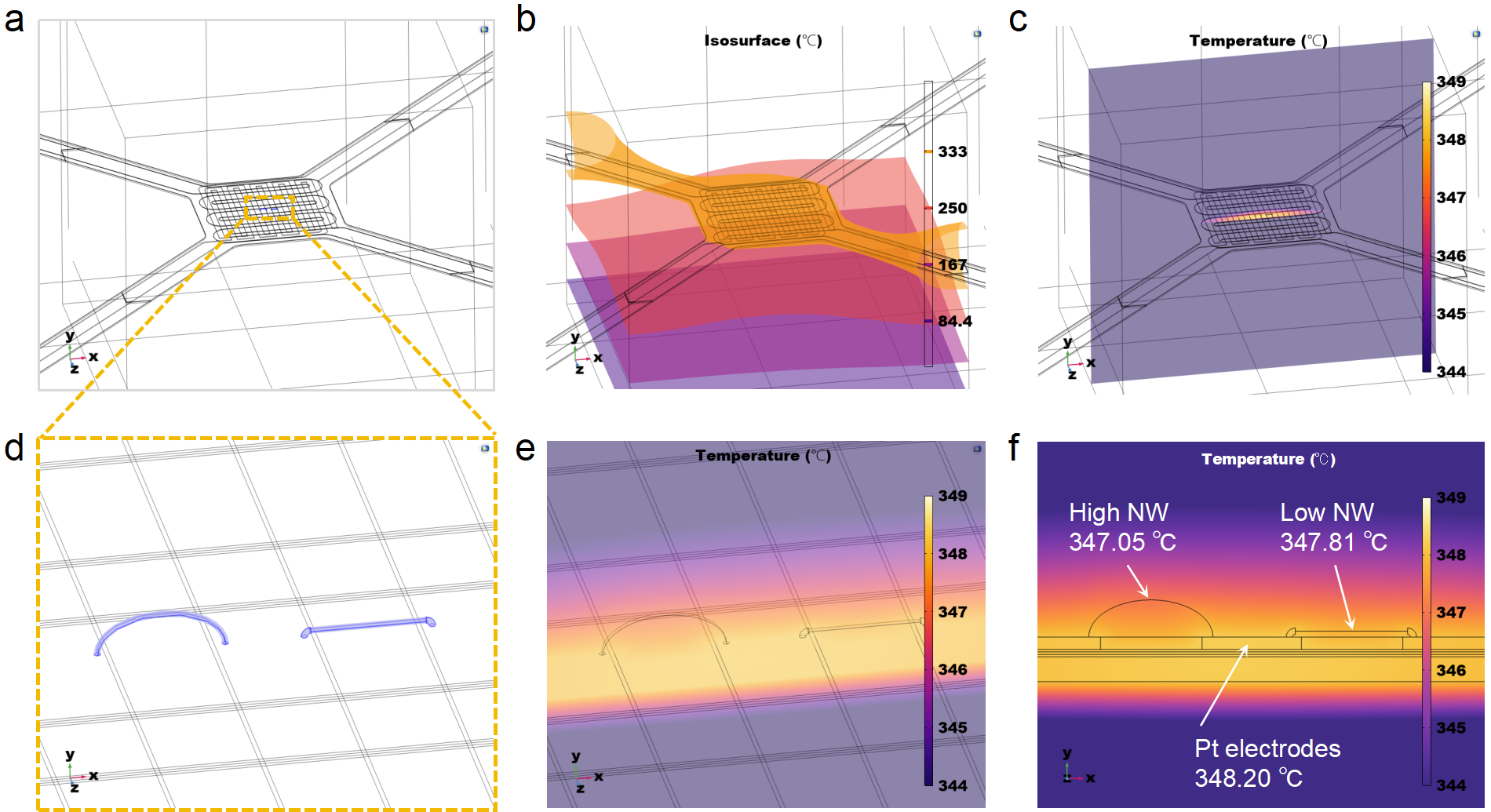


**Figure S10.** Numerical simulation of the temperature distribution of the freestanding nanowire sensor. (a) Schematic of the simulation model showing the MEMS device within an air domain. Thermal conductivity of the nanowire and membrane are set to 3 W/m·K and 20 W/m·K, respectively. (b) Isothermal surface plot visualizing the localized thermal envelope of heated air that forms around the MEMS heater. (c) Cross-sectional view showing the temperature profile across the solid MEMS device. (d) Top-down view of the two simulated freestanding nanowires with different arc heights. (e, f) Simulated temperature profile around the heater surface and the suspended nanowires.


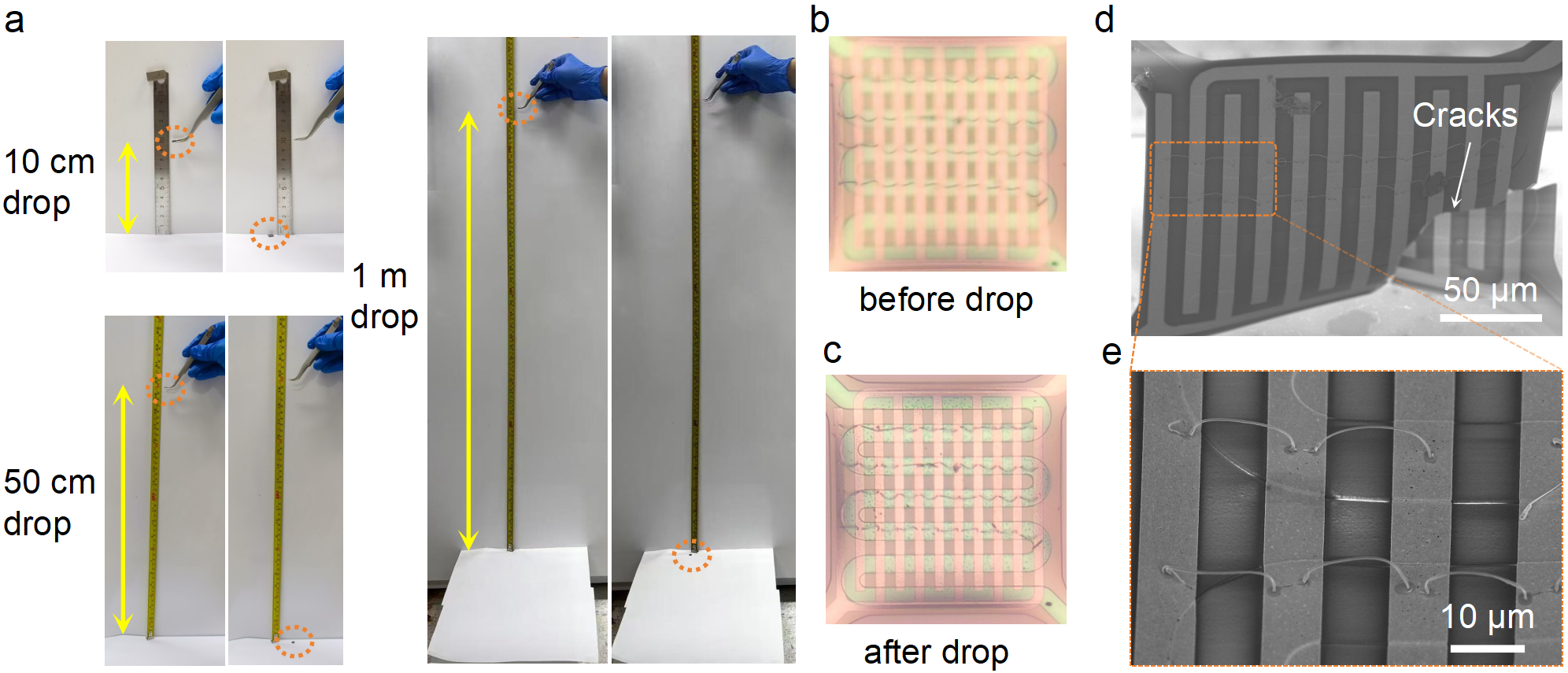


**Figure S11.** Drop test. (a-c). Optical microscopy images of the MEMS sensor before and after a 10 cm - 1 m drop onto a hard floor, demonstrating intact nanowire structures. (d). FE-SEM image of a MEMS device after the extreme impact mechanical fracture of the membrane. (e). Magnified FE-SEM image showing that the freestanding nanowires retained their arched morphology and structural integrity on the shattered membrane.


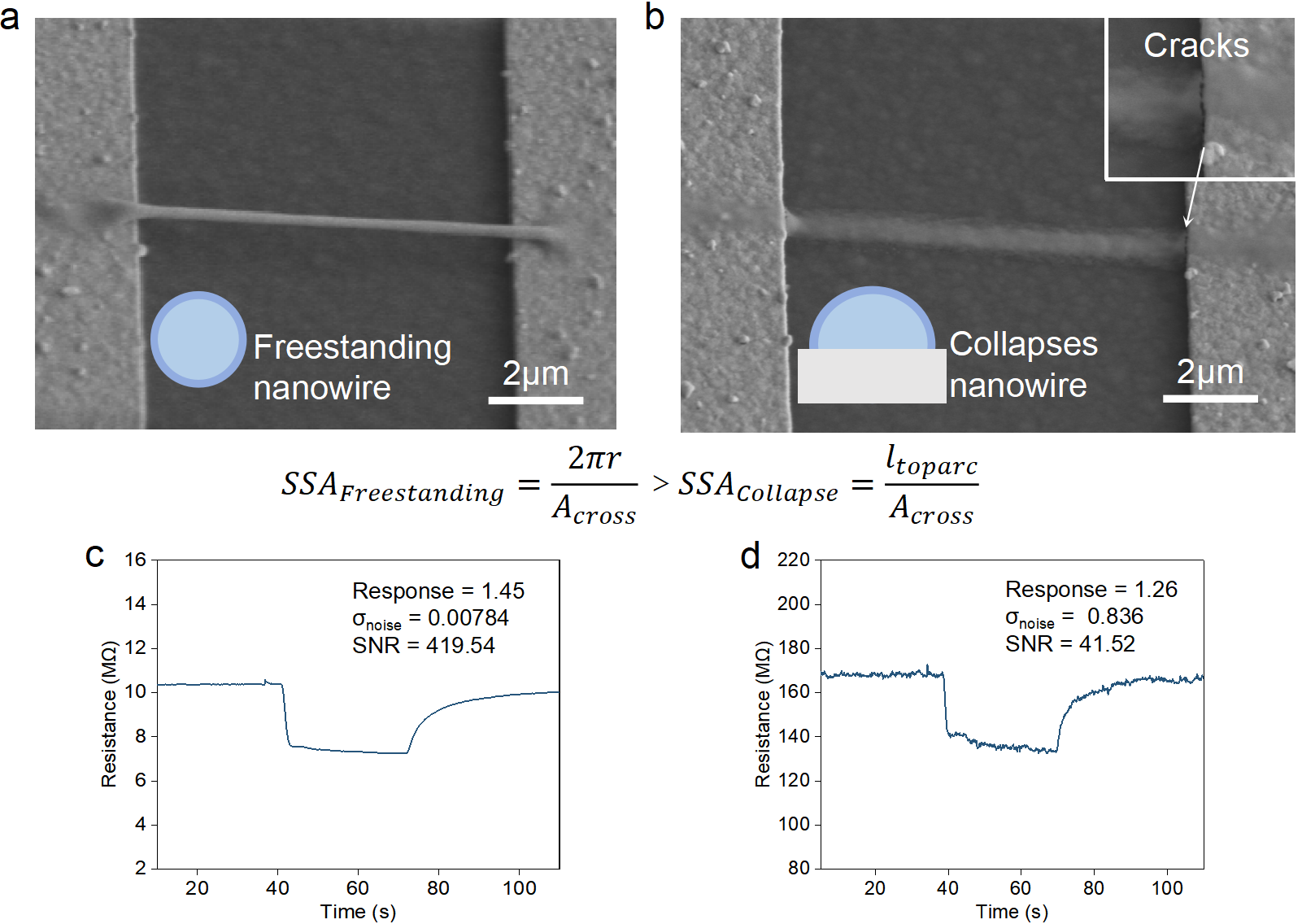


**Figure S12.** Comparison of SnO_2_/Pd freestanding nanowires and collapsed nanowires and their sensing performance to 10 ppm ethanol.


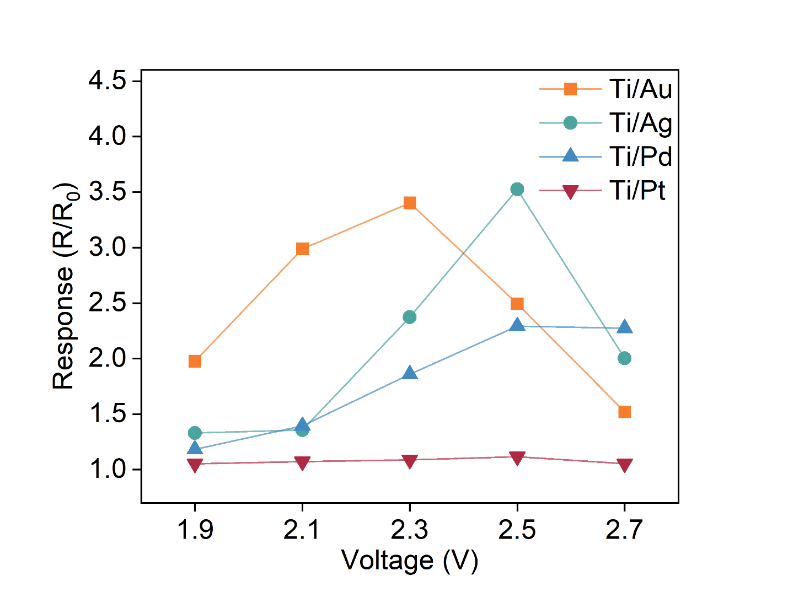


**Figure S13.** Four different gas sensors respond to 100 ppm NH_3_, depending on the heating voltage.


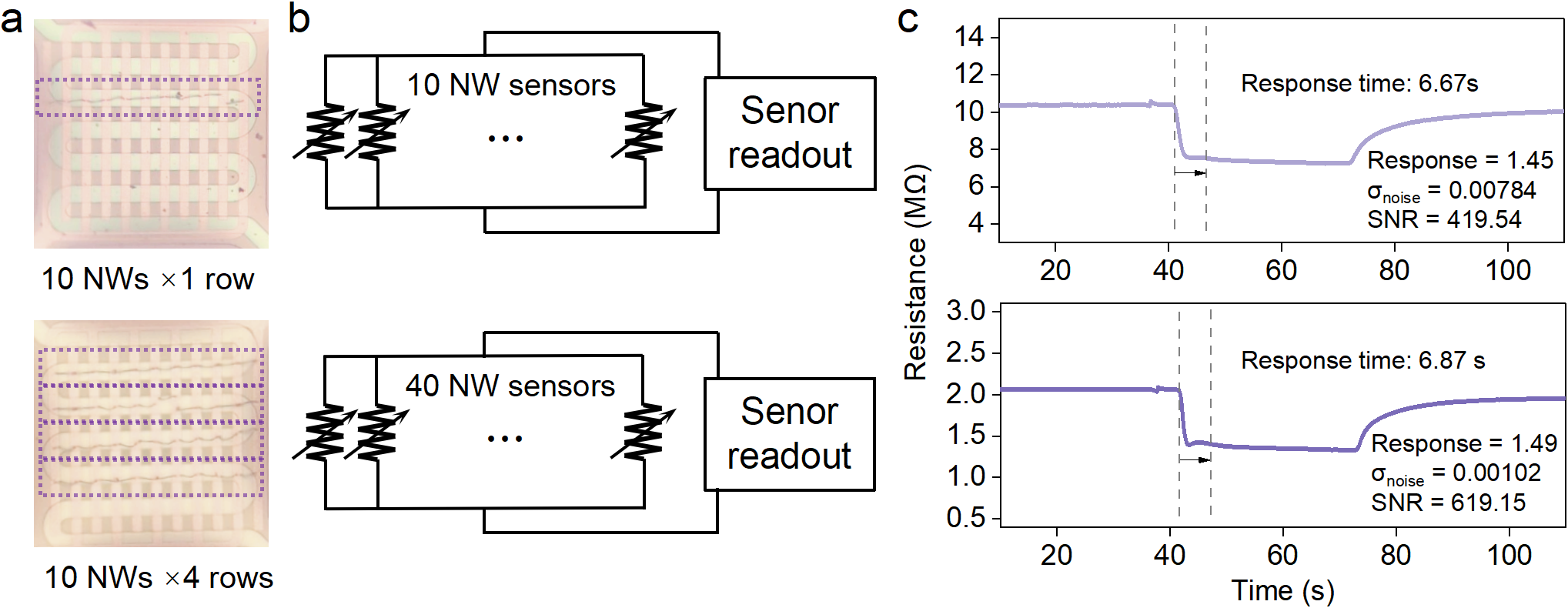


**Figure S14.** Comparison of the gas-sensing response of 10 nanowires and 40 nanowires integrated in parallel. (a) Optical micrographs, (b) electrical circuits, (c) resistive gas-sensing responses to 10 ppm ethanol. Increasing the number of nanowires aids gas-sensing performance by averaging out wire-to-wire variation and reducing the noise level.


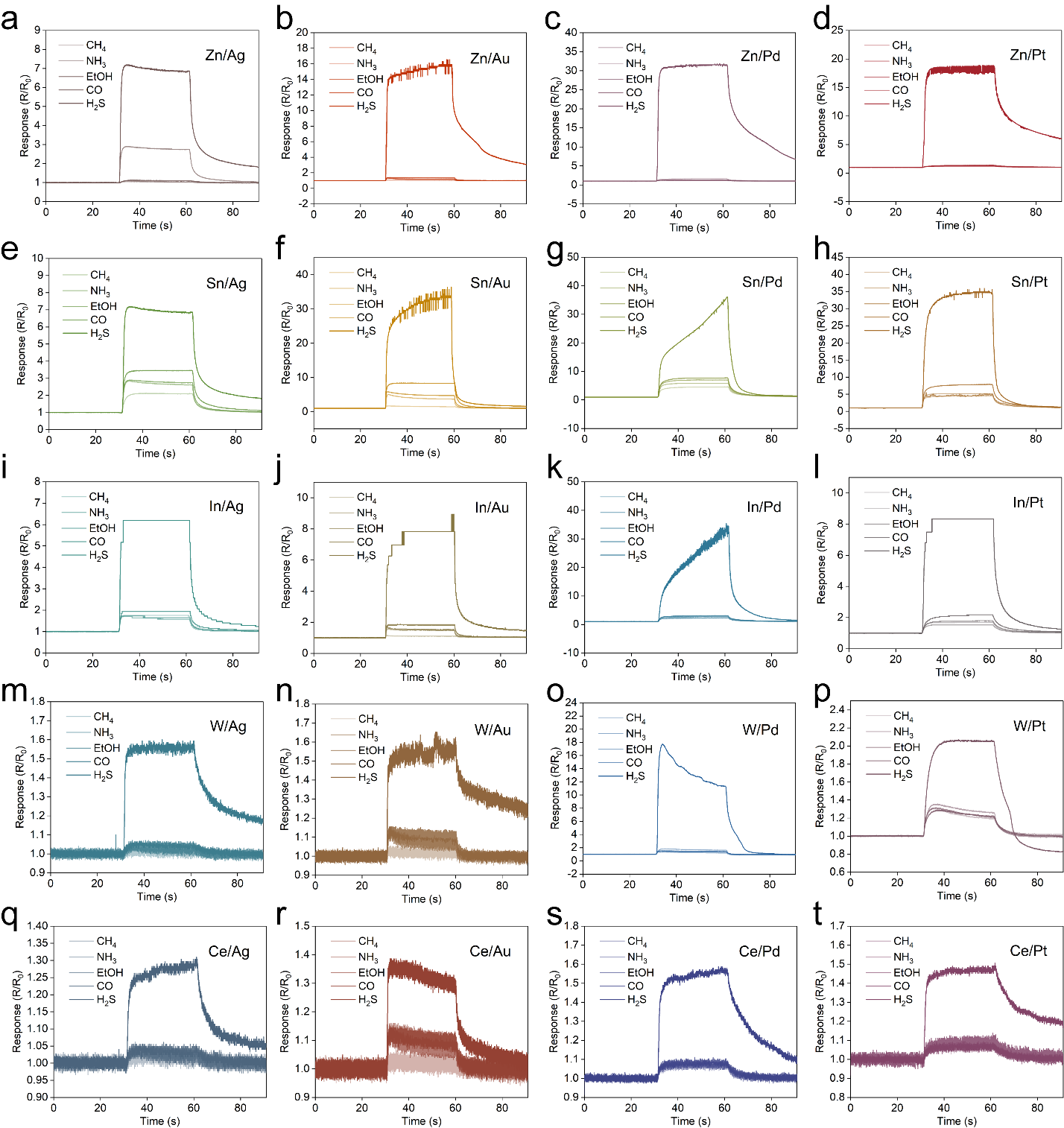


**Figure S15.** Real-time response-recovery curve of a) Zn/Ag, b) Zn/Au, c) Zn/Pd, d) Zn/Pt, e) Sn/Ag, f) Sn/Au, g) Sn/Pd, h) Sn/Pt, i) In/Ag, j) In/Au, k) In/Pd, l) In/Pt, m) W/Ag, n) W/Au, o) W/Pd, p) W/Pt, q) Ce/Ag, r) Ce/Au, s) Ce/Pd, and t) Ce/Pt nanowires sensor to five different gases of NH_3_, CH_3_CH_2_OH, CO, H_2_S, and CH_4_ at 100 ppm.


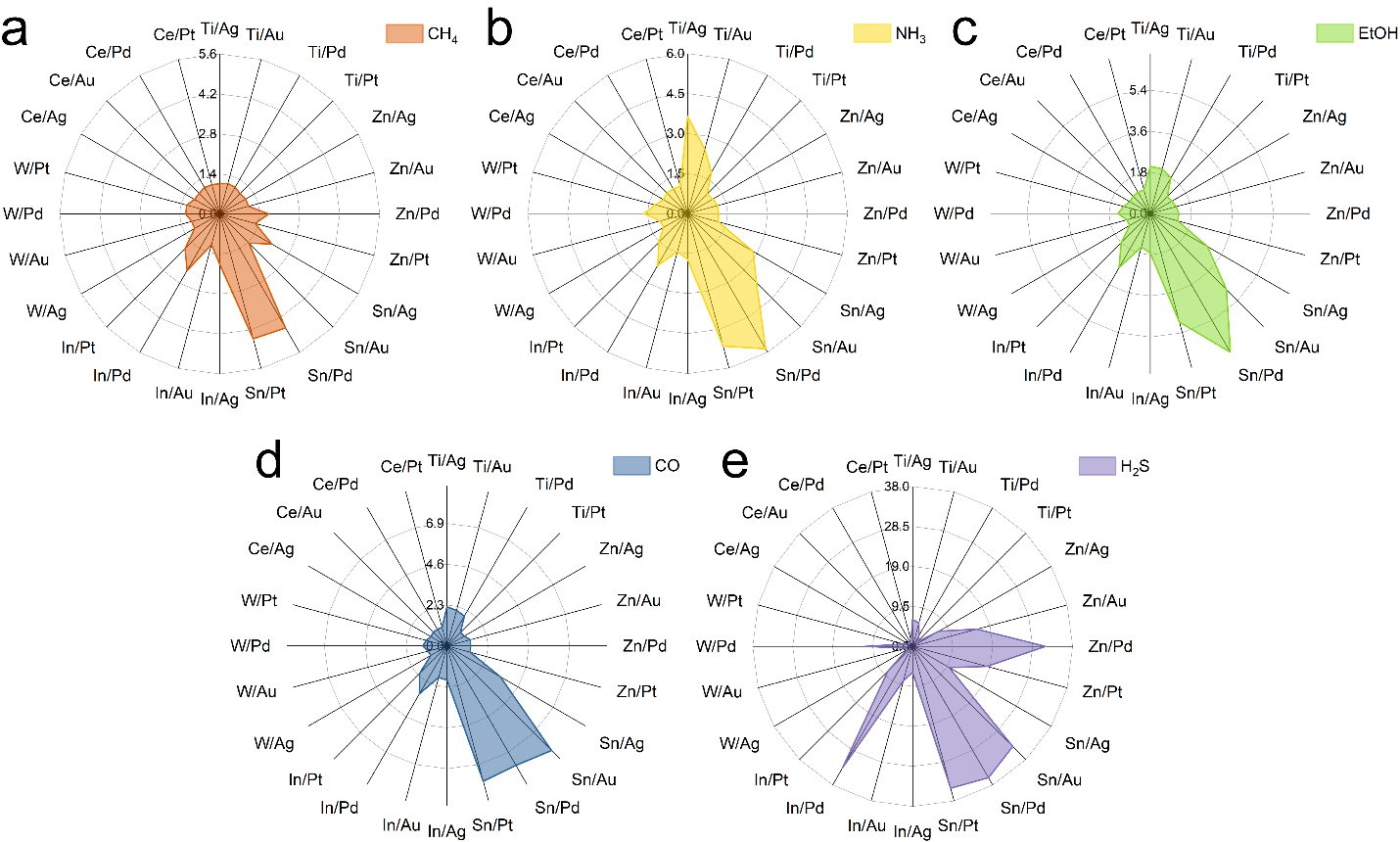


**FigureS16.** Radar chart patterns of the 24 types of nanowires for a) CH_4_, b) NH_3_, c) EtOH, d) CO, and e) H_2_S at 100 ppm.


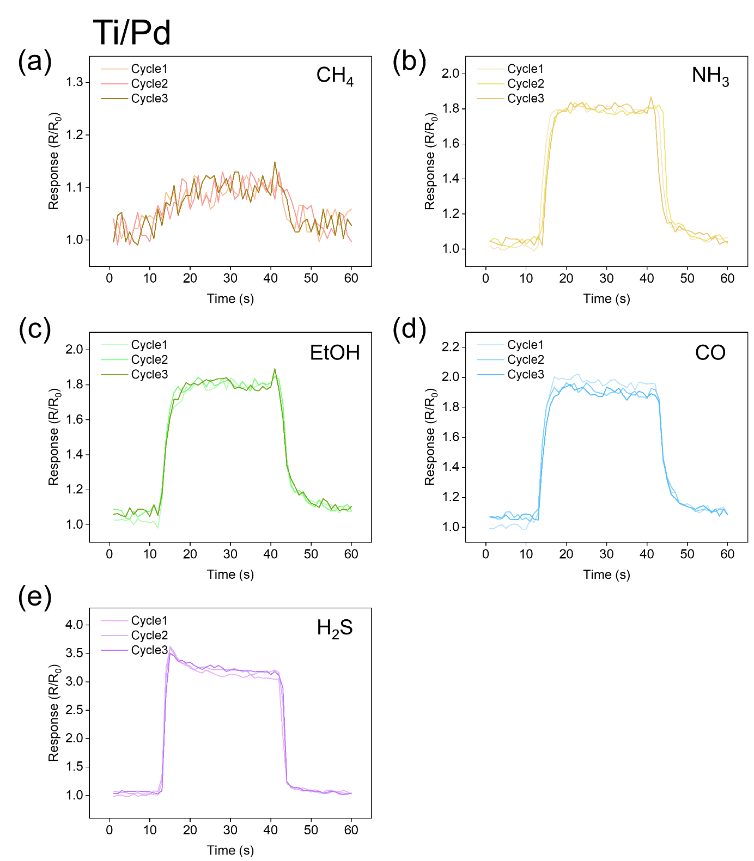


**Figure S17.** Repeated test results of Ti/Pd nanowire sensor for CH_4_, NH_3_, EtOH, CO, and H_2_S (Cycles 1, 2, and 3).


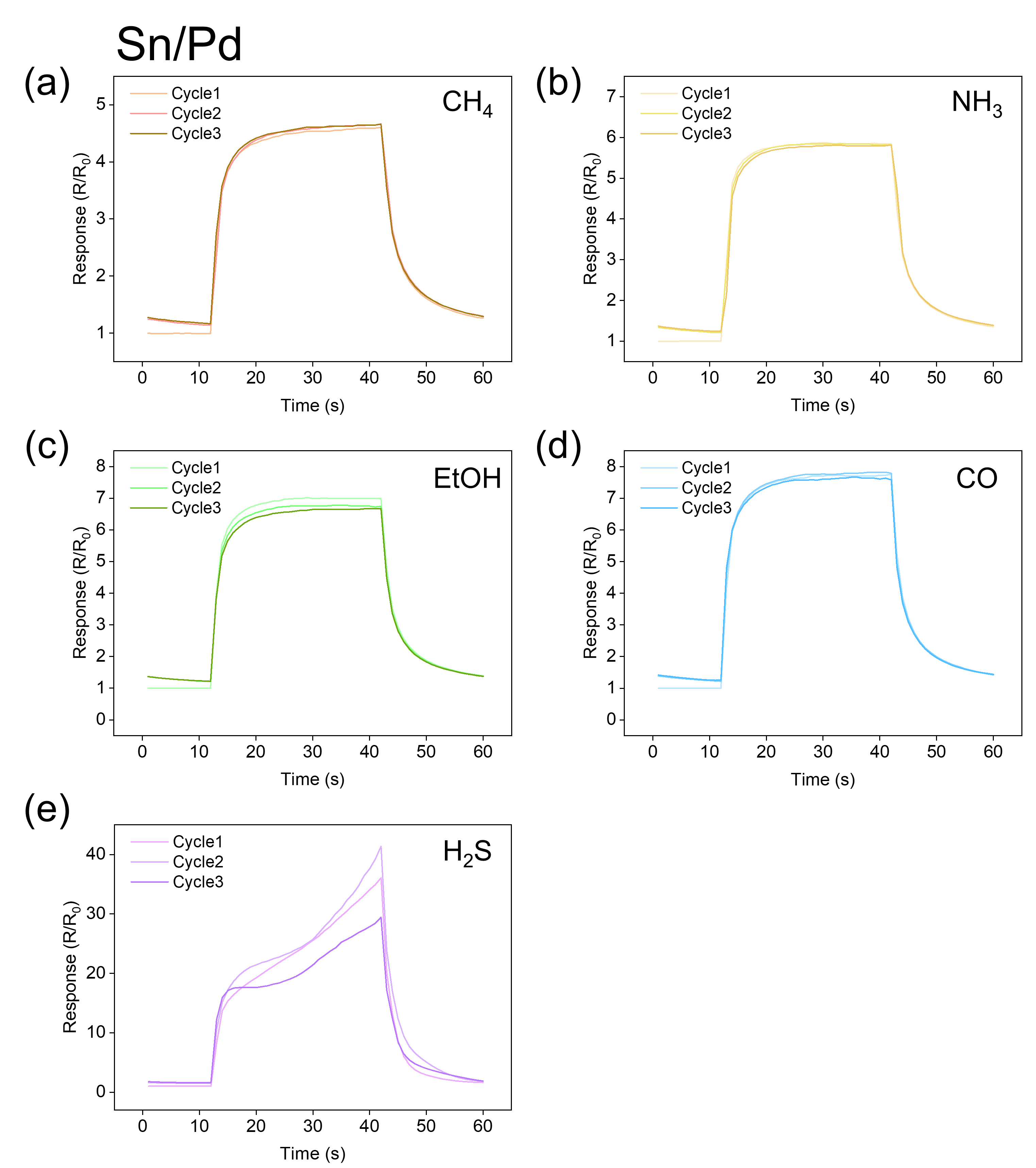


**Figure S18.** Repeated test results of Sn/Pd nanowire sensor for CH_4_, NH_3_, EtOH, CO, and H_2_S (Cycles 1, 2, and 3).

**Reference**

[1] H. Chen, J. Wang, S. Peng, D. Liu, W. Yan, X. Shang, B. Zhang, Y. Yao, Y. Hui, N. Zhou, *Nano-Micro Lett.* **2023**, 15, 180.

[2] H. Chen, X. Min, Y. Hui, W. Qin, B. Zhang, Y. Yao, W. Xing, W. Zhang, N. Zhou, *Mater. Horiz.* **2022**, 9, 764.

[3] T.-S. Kim, Y. Lee, W. Xu, Y. H. Kim, M. Kim, S.-Y. Min, T. H. Kim, H. W. Jang, T.-W. Lee, *Nano Energy* **2019**, 58, 437.

[4] C. Cao, X. Xia, X. Shen, X. Wang, Z. Yang, Q. Liu, C. Ding, D. Zhu, C. Kuang, X. Liu, *Nat. Commun.* **2024**, 15, 9216.

[5] H. Hu, C. Deng, H. Gao, T. Han, S. Xue, Y. Tang, M. Zhang, M. Li, H. Liu, L. Deng, W. Xiong, *Adv. Mater.* **2024**, 36, 2405053.

[6] B. Behera, S. Chandra, *Sens. Actuators, B* **2016**, 229, 414.

[7] Y. Nagarjuna, Y.-J. Hsiao, *Sens. Actuators, B* **2024**, 401, 134891.

[8] T. Zhang, R. Tan, W. Shen, D. Lv, J. Yin, W. Chen, H. Fu, W. Song, *Sens. Actuators, B* **2023**, 382, 133555.

[9] Z.-W. Zheng, S.-Y. He, Z.-Y. Huang, Y.-W. Wang, X.-F. Zhang, L.-X. Ou, D. W. Zhang, H.-X. Yu, H.-L. Lu, *Ceram. Int.* **2025**, 51, 31703.

[10] Y.-M. Yeh, S.-J. Chang, P.-H. Wang, T.-J. Hsueh, *Applied Sciences* **2022**, 12.

[11] Y. Chen, M. Li, W. Yan, X. Zhuang, K. W. Ng, X. Cheng, *ACS Omega* **2021**, 6, 1216.

[12] J. Zhao, W. Shen, Y. Gao, D. Lv, W. Song, R. Tan, *Sens. Actuators, A* **2025**, 391, 116680.

[13] Z. Chen, Y. Liu, R. Liu, Y. Chen, H. Liu, X. Cheng, *ACS Sens.* **2024**, 9, 3979.

[14] I. Cho, K. Kang, D. Yang, J. Yun, I. Park, *ACS Appl. Mater. Interfaces* **2017**, 9, 27111.

[15] Q. Luo, R. Liu, Y. Li, Y. Li, K. Wang, Y. Liu, J. Sun, K. Huang, L. Zhong, J. Wang, X. Cheng, *Sens. Actuators, B* **2025**, 439, 137854.

[16] Y. Zhang, C. Zhang, Z. Zhang, H. Zong, P. Tan, L. Luo, Y. Luo, G. Duan, *Small* **2025**, 21, 2411422.

[17] D. Im, D. Kim, D. Jeong, W. I. Park, M. Chun, J.-S. Park, H. Kim, H. Jung, *Journal of Materials Science & Technology* **2020**, 38, 56.

[18] P. H. Wen, H. Y. Zheng, T.-J. Hsueh, *J. Electrochem. Soc.* **2023**, 170, 037506.

[19] Z. Dong, Q. Hu, H. Liu, Y. Wu, Z. Ma, Y. Fan, R. Li, J. Xu, X. Wang, *Sens. Actuators, B* **2022**, 357, 131227.

[20] Q. Duan, W. Zhang, L. Li, B. Ying, T. Li, B. Chen, H.-Y. Li, H. Liu, *Chem. Eng. J.* **2024**, 479, 147748.
